# Surface chemistry modified upconversion nanoparticles as fluorescent sensor array for discrimination of foodborne pathogenic bacteria

**DOI:** 10.1186/s12951-020-00596-4

**Published:** 2020-02-28

**Authors:** Mingyuan Yin, Chuang Jing, Haijie Li, Qiliang Deng, Shuo Wang

**Affiliations:** 1 State Key Laboratory of Food Nutrition and Safety, School of Food Engineering and Biotechnology, College of Chemical Engineering and Materials Science, Tianjin University of Science and Technology, Tianjin, 300457 People’s Republic of China; 2 Tianjin Key Laboratory of Food Science and Health, School of Medicine, Nankai University, Tianjin, 300071 People’s Republic of China

**Keywords:** Upconversion nanoparticles, Foodborne bacteria, Sensor array, Identification

## Abstract

**Background:**

The identification of foodborne pathogenic bacteria types plays a crucial role in food safety and public health. In consideration of long culturing times, tedious operations and the desired specific recognition elements in conventional methods, the alternative fluorescent sensor arrays can offer a high-effective approach in bacterial identification by using multiple cross-reactive receptors. Herein, we achieve this goal by constructing an upconversion fluorescent sensor array based on anti-stokes luminogens featuring a series of functional lanthanide-doped upconversion nanoparticles (UCNPs) with phenylboronic acid, phosphate groups, or imidazole ionic liquid. The prevalent spotlight effect of microorganism and the electrostatic interaction between UCNPs and bacteria endow such sensor array an excellent discrimination property.

**Results:**

Seven common foodborne pathogenic bacteria including two Gram-positive bacteria (*Staphylococcus aureus* and *Listeria monocytogenes*) and five Gram-negative bacteria (*Escherichia coli*, *Salmonella*, *Cronobacter sakazakii*, *Shigella flexneri* and *Vibrio parahaemolyticus*) are precisely identified with 100% accuracy via linear discriminant analysis (LDA). Furthermore, blends of bacteria have been identified accurately. Bacteria in real samples (tap water, milk and beef) have been effectively discriminated with 92.1% accuracy.

**Conclusions:**

Current fluorescence sensor array is a powerful tool for high-throughput bacteria identification, which overcomes the time-consuming bacteria culture and heavy dependence of specific recognition elements. The high efficiency of whole bacterial cell detection and the discrimination capability of life and death bacteria can brighten the application of fluorescence sensor array.

## Background

The detection and discrimination of foodborne pathogenic bacteria is a crucial issue in environmental monitoring, food safety and early diagnosis of diseases [1–5]. Every year over 300 million illnesses and more than 5 million deaths result from pathogenic bacterial infection, causing inestimable loss of property [6]. Therefore, the constant threats from existing and emerging foodborne pathogenic bacteria make the rapid and reliable identification and quantification of bacterial species an important index for shriveling foodborne pathogenic bacteria contamination [3, 7].

Up to present, the extensively adopted methods for bacterial identification include plate cultivation, morphological structure observation, gene and immunological characteristics analysis [4, 6]. However, the traditional bacterial species identification methods heavily rely on time-consuming and low-accuracy phenotypic characterizations such as serotype by Gram staining and biochemical methods [4]. Polymerase chain reaction (PCR), gene sequencing, surface enhanced Raman scattering (SERS), and mass spectrometry as well as a series of specificity sensing methods for analysis bacteria based on the affinity reagents (including peptide, phage, antibiotic, antibody, and aptamer) have been widely employed to precise traceability analysis [8–13], the main limitations of these methods are highly reliant on expensive reagents, sophisticated costly and bulky equipment, elusive manipulations, and trained personnel and handling conditions [7, 13]. Furthermore, to ensure effective treatments, timely and reliable diagnosis of pathogen infection is the primary step [14]. Lacking of reliable and punctual pathogenic contamination information, the suboptimal selective drug treatment can delay the best treatment timing and lead to strains undergo mutations and acquire antibiotic resistance [6, 12]. A dire prediction shows that 10 million people can be killed by antibiotic-resistant bacteria infections worldwide by 2050 [6]. Thus, developing timely and efficient methods to discern foodborne pathogenic bacteria are urgent need for food security and public health [7, 12, 13].

The fluorescent probe is an alternative and promising tool for the identification of pathogenic bacteria with great temporal and spatial sampling capability, rapid response, and high sensitivity and simplicity [15–18]. The biosensor for pathogenic bacteria identification based on fluorescent responses has received more and more attention [16, 19]. However, conventional luminescence elements generally convert high-energy photons into low-energy photons (such as quantum dot, fluorescent conjugated polymer, and fluorochrome) [20], and the photobleaching and background noise as well as ground substance disturbance are still inescapable [6]. This has led to their narrow scope of application, low level working concentration and weak labeling degrees to analyte, generating compromised sensitivity and accuracy of identification [6]. To tackle these issues, the fluorescence probes for bacterial identification based on the conventional sensing elements will be thoroughly covered by lanthanide-doped upconversion nanoparticles (UCNPs) materials with anti-stokes luminescence [10–12, 21].

Diametrically opposed to the conventional luminescence sensing elements, UCNPs materials are able to convert low-energy photons (such as near-infrared light) into high-energy photons (such as visible and ultraviolet light), which is ideal for sensing analyte in complex environment [22–24]. Moreover, UCNPs-based probes have excellent photo-stability, multicolor tunable property, less toxic elements, negligible autofluorescence background and the minimal photo blinking and photo bleaching [10–12, 21, 25], which can heavily improve the sensitivity and reliability of detection. In addition, surface chemistry modification further endows UCNPs materials versatile properties and enables these functional UCNPs materials to realize the tunable charges and solubility, and target diversity, which provides a large library for sensor selection [10, 12, 22, 26, 27].

Recently, the fluorescent sensor arrays constructed from a series of functioned UCNPs probes have exhibited excellent power for identification of analytes (such as phosphate compounds, grape wines, proteins, glycated hemoglobin, vitamin B12, viruses, biotinylated antibodies and human IgG and IgM antibodies) with high classification accuracy in a timely and cost-effective manner [22, 28–33]. UCNPs materials have been widely utilized to realize the quantification of single bacteria in previous researches [9–12, 34]. In addition, multicolor upconversion nanoparticles modified with specific recognition element (aptamers and antibody) have also been constructed for the detection of multiplex pathogenic bacteria [35, 36].

In this research, we introduce the multivalent interactions between UCNPs materials and bacteria to improve the fluorescence response towards bacteria, taking advantage of the prevalent spotlight effect of microorganism as well as the electrostatic interaction [10, 12]. Three surface chemistry modification UCNPs probes (bearing one positively charged imidazole ionic liquid groups, two negatively charged phenylboronic acid or phosphate groups) are designed and synthesized. A fluorescent sensor array composed of the three UCNPs probes is constructed, which can successfully distinguish seven representative foodborne pathogenic bacteria (*Escherichia coli* (*E. coli*), *Staphylococcus aureus* (*S. aureus*), *Salmonella*, *Listeria monocytogenes* (*L. monocytogenes*), *Cronobacter sakazakii* (*C. sakazakii*), *Shigella flexneri* (*S. flexneri*) and *Vibrio parahaemolyticus* (*V. parahaemolyticus*)) through pattern recognition with linear discriminant analysis (LDA) and realize the bacterial analyses from real samples (tap water, milk and beef).

## Materials and methods

### Materials

Lanthanide acetate hydrates (99.9%, Ln (Ac)_3_, Ln = Y, Yb, and Er) were obtained from Alfa Aesar Co. Ltd. (Ward Hill, MA, USA). Oleic acid (OA, 90%), 1-octadecene (ODE, 90%), tetraethyl orthosilicate (TEOS, 98%), allyltriethoxysilane (ATS, 97%), 1-vinylimidazole (VID, 99%) and 1-bromooctane (99%) were obtained from J&K Chemical (Beijing, China). Vinylphosphonic acid (VPA, 97%) and 4-vinylphenylboronic acid (VPBA, 95%) were purchased from Sigma Aldrich (St. Louis, MO, USA). Triton X-100 was supplied by GFCO Chemical (Hongkong, China). Azodiisobutyronitrile (AIBN, 98%), ethanol (95%), cyclohexane (95%) and ammonia solution (25%) were brought from North Tianyi Chemical Reagent Factory (Tianjin, China). Luria–bertani (LB) broth, agar powder, nutrient agar/broth, brain heart infusion broth, dimethyl sulfoxide (DMSO) and phosphate buffer saline (PBS) were ordered from Beijing Solarbio Science & Technology Co., Ltd. (Beijing, China). Double distilled water (DDW, 18.2 MΩ cm^−1^) was produced by a Water Pro water purification system. *E. coli* (ATCC25922), *S. aureus* (ATCC25923), *Salmonella* (CICC10867), *L. monocytogenes* (ATCC7644), *C. sakazakii* (ATCCBAA894), *S. flexneri* (ATCC12022) and *V. parahaemolyticus* (ATCC17802) were ordered from BeNa Culture Collection Co., Ltd. (Beijing, China). Milk and beef were purchased from the local supermarket. Tap water was collected from water pipe in Tianjin University of Science and Technology campus. Other reagents were at least of analytical grade and without further purification.

### Characterization

Fluorescence spectra were measured on an F-7000 fluorescence spectrometer (Hitachi, Tokyo, Japan) attached with an external 980 nm laser (2 W, continuous wave with 1 m fiber, Beijing, China) instead of internal excitation source. Transmission electron microscopy (TEM, 200 kV) images were obtained on a JEOL 2010F (JEOL, Japan) with an attached energy-dispersive X-ray spectroscope (EDS). Fourier transform infrared (FT-IR) spectra (4000–400 cm^−1^) in KBr were recorded in a Vector 22 FT-IR spectrophotometer (Bruker, Karlsruhe, Germany). The zeta potentials of UCNPs materials were measured at room temperature in neutral water solution with a Zetasizer Nano ZS90 (Malvern, Worcestershire, U.K.). Ultraviolet–Visible (UV–Vis) absorbance spectra were recorded on a Shimadzu UV-2700 UV–Vis spectrophotometer (Shimadzu, Japan). The suspension of nanoparticles was prepared using an ultrasonic bath SBL-10DT (Ningbo, China). Thermogravimetric analysis (TGA) was carried out by STA 449 F5 Jupiter Netzsch thermogravimeter (Netzsch, Selb, Germany).

### Synthesis of UCNPs@COPs materials

The oil–solvent lanthanide-doped UCNPs and the co-polymers (COPs) were first prepared as procedure described in Additional file 1. The UCNPs@COPs materials were obtained by inverse microemulsion method as following, UCNPs (0.04 mM) were dissolved into acetonitrile (6.0 mL) containing Triton X-100 (0.1 mL) under stirring. Next, ammonia solution (0.08 mL) and Triton X-100 (0.4 mL) were added and sonicated for 20 min. Subsequently, TEOS (40 µL) and the as-prepared COPs (0.04 mM) were slowly dropped in sequence. After the system was stirred at room temperature for 24 h, UCNPs@COPs were collected and washed with ethanol for several times.

### Bacteria culturing and counting

Seven representative foodborne pathogenic bacteria including *E. coli*, *S. aureus*, *Salmonella*, *L. monocytogenes*, *C. sakazakii*, *S. flexneri* and *V. parahaemolyticus* were obtained as previous process [12]. The obtained bacteria were centrifuged at 3000*g* for 5 min, washed with physiological saline solution for three times, and diluted to the desired concentration. The number of bacteria (OD_600_ = 0.1) were estimated by the plate counting method (Additional file 1: Table S2). The death *E. coli* and *S. aureus* were obtained by ultrasonic treatment. Tubes containing 1 mL aliquots of bacteria suspension (OD_600_ = 0.5) were treated by ultrasonic for 20 min (The ultrasonic operates at a nominal frequency of 40 kHz).

### Optimization of sensor array working conditions

In order to obtain the optimal detection conditions, *E. coli* (OD_600_ = 0.5) was chosen as a model bacterium. The fluorescence spectra of the different concentrations of the three UCNPs@COPs materials (0.05, 0.10, 0.15, 0.20, 0.25, and 0.30 mg mL^−1^) treated with *E. coli* were recorded. The optimum incubating time of the three UCNPs@COPs materials treated with *E. coli* was investigated by measuring the fluorescence emission intensity at certain intervals (2, 4, 6, 10, 20, 25, 30, 35, and 60 min).

### Bacterial identification based on sensor array

Generally, the solutions of the three UCNPs@COPs (0.20 mg mL^−1^) were prepared in physiological saline with 10% DMSO, and mixed with bacterial solutions (v/v = 1:1). Subsequently, the fluorescence spectra of the mixtures were recorded. The fluorescence emission intensity at 550 nm of each UCNPs@COPs material for each bacterial species was collected, and the training set was obtained by selecting six independent replicates.

### Fluorescence response patterns

The collected date of fluorescence response patterns ((F_i_-F_0_)/F_0_) were analyzed using SPSS (IBM SPSS Statistics 22), and the raw data matrix (3 × UCNPs@COPs × 7 bacteria × 6 replicates) was processed using the linear discriminant analysis (LDA) and hierarchical clustering analysis (HCA). For classification, the training set was used to perform LDA, obtaining the corresponding scores as well as coefficients. For HCA, the average linkage method was adapted.

### Identification of bacteria in real sample

As common sources of bacterial contamination, tap water, milk and ground beef were selected as real samples. Standard addition method with a series of pre-treatment procedure was performed referencing to the previous report [36]. For the tap water (following the sanitary standards for drinking water quality of the state standard of the People's Republic of China (GB 5749-2006) [37]), sample (100.0 mL) was collected from water pipe without any pre-treatment and added the desired concentration standard bacterial stock for the following detection. Specifically, for the milk sample (about 6% protein content, 7% fat content and 15% calcium content), the desired concentration standard bacterial stock was added to 25.0 mL of aseptic sample. After centrifugation at 7000*g* for 10 min to remove the upper layer of cream, the milk sample was then diluted with sterile water (1:9). For the ground beef sample (about 85% lean content and 15% fat content), sample (25.0 g) were added the desired concentration of standard bacterial stock and homogenized for 5 min in 225.0 mL of aseptic 1 × PBS. Then, the solution was maintained for 30 min to precipitate macroaggregates and seston. Finally, the supernatant was collected through a 0.45 µm filtration membrane. After these pre-treatment steps, both the spiked samples and raw samples (as control) were subjected to UCNP@COPs probes following the general procedure. And the total acquisition time of the bacterial detection in different samples was estimated to 15 min (tap water), 30 min (milk), and 60 min (beef).

## Results and discussion

### Characterization of UCNPs materials

**Fig. 1:**
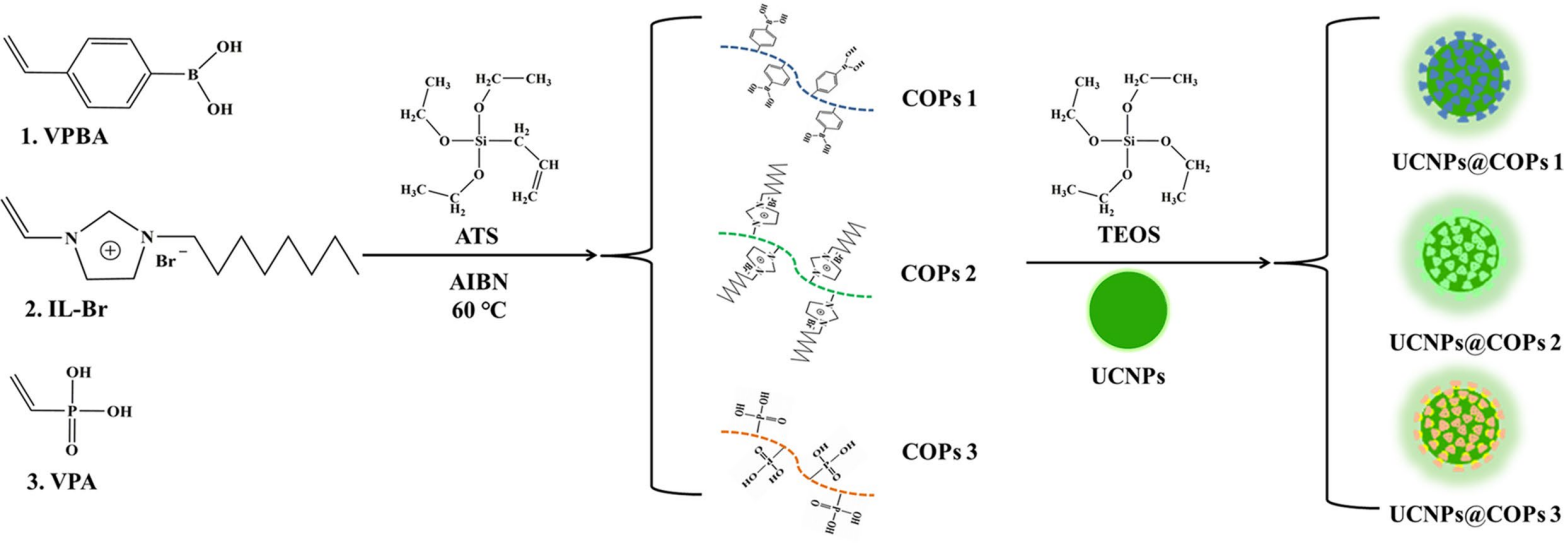
Synthesis of UCNPs@COPs materials

In order to develop UCNPs-based materials for bacteria recognition, three surface chemistry modification versatile UCNPs materials with functional groups including phenylboronic acid (UCNPs@COPs 1), imidazole ionic liquid (UCNPs@COPs 2), and phosphate groups (UCNPs@COPs 3) were designed as sensing probes. As shown in the Fig. 1 and Additional file 1: Table S1, allyltriethoxysilane (ATS) and functional monomers (4-vinylphenylboronic acid (VPBA), 1-octyl-3-vinylimidazolium bromide ionic liquid (IL-Br) and vinylphosphonic acid (VPA)) were employed to pre-compose cross-linked COPs via free-radical polymerization triggered by AIBN, then UCNPs@COPs materials were obtained by grafting COPs onto the surface of UCNPs via inverse microemulsion method.

The elemental analyses were first carried out by EDS to confirm the composition of UCNPs materials (Additional file 1: Figure S1). Common elementals of F, Na, Y, Yb, and Er were observed in all the obtained UCNPs@COPs materials. Additionally, the characteristic elementals such as B in UCNPs@COPs 1, N in UCNPs@COPs 2, and P in UCNPs@COPs 3 were emerged, respectively, indicating that COPs were successfully grafted onto UCNPs.

**Fig. 2:**
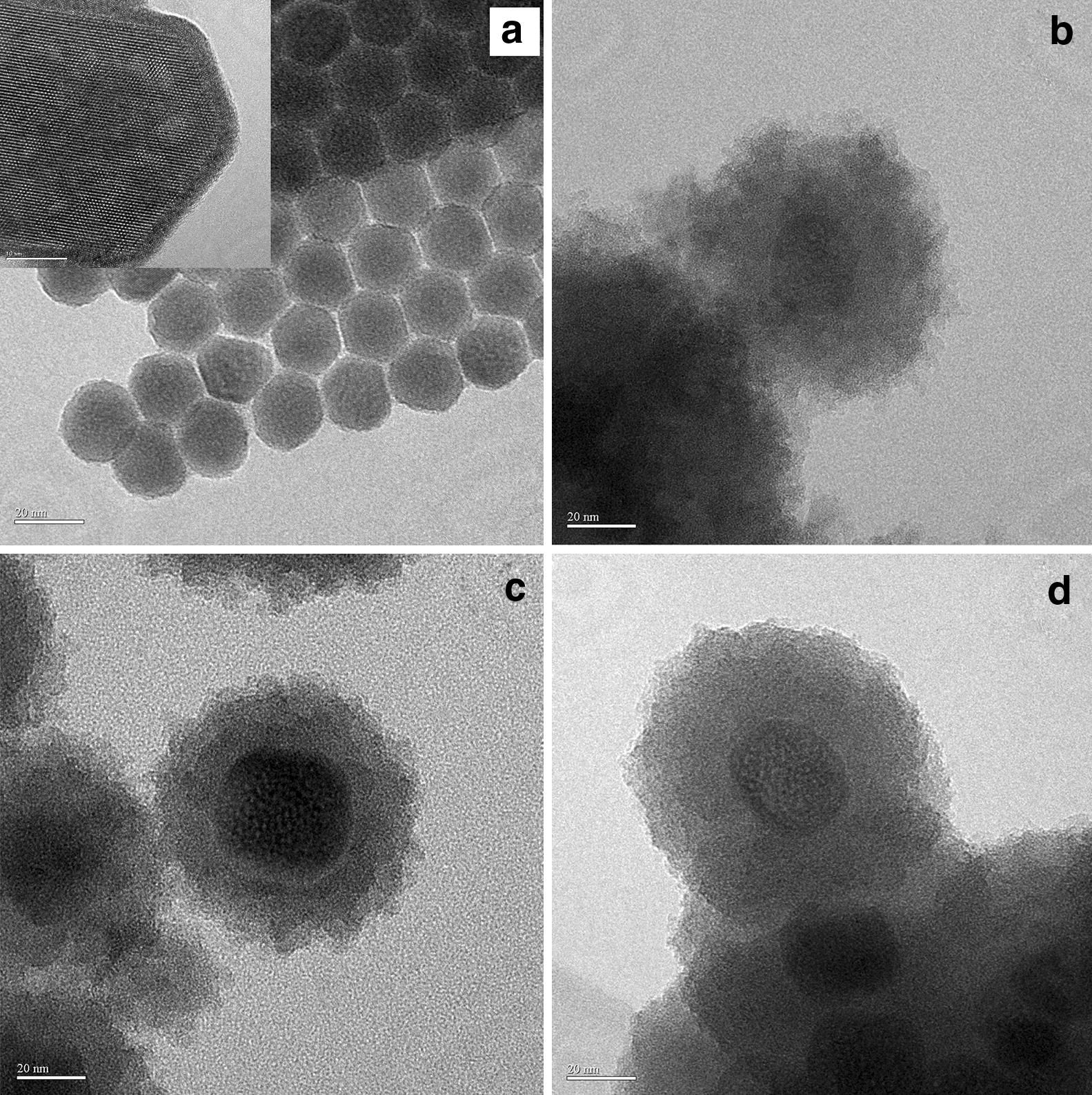
TEM images of UCNPs (**a**), UCNPs@COPs 1 (**b**), UCNPs@COPs 2 (**c**) and UCNPs@COPs 3 (**d**)

The investigation of TEM images exhibited that monodisperse hexagonal nanoprisms of UCNPs with a uniform particle size about 20 nm was formed, and the COPs layers (around 20 nm thickness) have been coated onto the surface of UCNPs (Fig. 2). Although the morphologies of the three UCNPs@COPs materials exhibited discrepant core–shell structure, it was not studied in detail because the reshaping did not effect on the fluorescence properties. XRD analysis (Additional file 1: Figure S2) revealed that the position and peak shape of major diffraction peaks of UCNPs@COPs in 2θ were good consistent with the bare UCNPs and the standard alignment card (JCPDS standard card number 16-0334), indicating that all the materials were equipped with β-phase crystal. And no impurity diffraction peak was found in UCNPs@COPs, indicating that the hexagonal-phase structure was not disturbed by the COPs layers.

Compared with FITR spectra of UCNPs, the most characteristic bands of UCNPs@COPs materials revealed significant changes in the band intensity and vibration frequency (Additional file 1: Figure S3). The characteristic peaks of UCNPs at 1419 and 1560 cm^−1^ were attributed to asymmetric and symmetrical stretching vibration peaks of carboxyl groups of OA molecules, and the obvious peaks at 2854, and 2926 cm^−1^ raised from the stretching vibration peak of the methyl and methylene groups of OA molecule. Grafting COPs on the surface of UCNPs, the characteristic peaks of OA molecule diminished or disappeared. Meanwhile, the stretching vibration characteristic peaks of Si–O–Si were observed at 1085, 1087, and 1091 cm^−1^ in the three UCNPs@COPs materials. In addition, the characteristic peaks at 1387 and 1636 cm^−1^ belonged to the stretching vibration peaks of B-O and benzene ring structure of UCNPs@COPs 1. The characteristic peaks at 1504 and 1633 cm^−1^ raised from the stretching vibration of imidazole ring structure of UCNPs@COPs 2. The characteristic peaks at 1389 and 1627 cm^−1^ were attributed to the stretching vibration peaks of P=O and P–O of UCNPs@COPs 3. Triton X-100 was involved during the preparation of the materials. Therefore, the removal of such compound on the particle surface was investigated via the monitoring of UV–Vis absorbance spectra. As shown in Additional file 1: Figure S4A, the key ultraviolet absorbance peaks of triton X-100 were significantly observed in the pre-treated UCNPs@COPs, however, disappeared in the post-treated UCNPs@COPs. In addition, we also checked Triton X-100 in the washing solution. As shown in Additional file 1: Figure S4B, the concentration of Triton X-100 reduced as the washing times increase. Consistent with previous study [38], all these results indicated that Triton X-100 was removed from UNCPs materials via washing with ethanol.

Thermal stabilities of the three UCNPs@COPs materials were further characterized by the thermogravimetric analysis (TGA). As shown in Additional file 1: Figure S5, the first weight loss of the three UCNPs@COPs materials occurred around 100 °C, which was arisen from the desorption of atmospheric moisture. The second thermal decomposition observed in the range of 160–400 °C was arisen from the dehydrations of UCNPs@COPs materials. Final thermal decomposition (in the range from 400 to 650 °C) was attributed to the decomposition of the COPs layers. From 650 to 800 °C, thermal decomposition became neglected, and the weight losses of UCNPs@COPs 1, 2, and 3 were steady decline to 17%, 19% and 16%, respectively, which were more than that of the bare UCNPs (11%). TGA results revealed the good thermal stabilization of UCNPs@COPs materials.

Zeta potential is a significant factor for fluorescence probe, and the three UCNPs@COPs materials revealed significant difference in terms of surface potentials. As shown in Additional file 1: Figure S6, the bare UCNPs displayed the positive surface potentials (+ 3.85 mV), and the surface potentials of UCNPs@COPs 1, UCNPs@COPs 2, and UCNPs@COPs 3 were − 17.13 mV, + 31.93 mV, and − 30.63 mV, respectively. All these results further confirmed that the COPs have been successfully modified onto the surface of UCNPs materials.

As an excellent anti-stokes luminescent element, the fluorescence emission spectra of the three UCNPs@COPs materials under 980 nm excitation wavelengths were further investigated in physiological saline solution/DMSO (9:1). As expected, the prominent fluorescence emission spectra of the three UCNPs@COPs materials showed the typical green upconversion emissions of NaYF_4_: Er^3+^, Yb^3+^ (the characteristic of Er^3+^ corresponded to the (^2^H11/2 and ^4^S3/2) → ^4^I15/2 transitions) [27], and the fluorescence emission intensity of the three UCNPs@COPs materials decreased slighter than that of the bare UCNPs at the same concentration, which was attributed to the weakened luminescence caused by the capping agents COPs (Additional file 1: Figure S7A). Meanwhile, the capping agents COPs with different functional groups did give rise to certain difference of fluorescence emission intensity. Although the fluorescence emission intensities of UCNPs@COPs probes were weaker than that of the bare UCNPs materials, the fluorescence responses of all the three UCNPs@COPs probes caused by the alive *E.coli* were more sensitivity than that of the bare UCNPs, which demonstrated the COPs layer had a crucial role for the bacteria binding (Additional file 1: Figure S7B). All above results have demonstrated that the three UCNPs@COPs materials not only inherited the good luminescence properties of UCNPs materials, but also gave abundant action sites, which could be ideal materials in the sensing field.

### Role of UCNPs@COPs materials

**Scheme 1:**
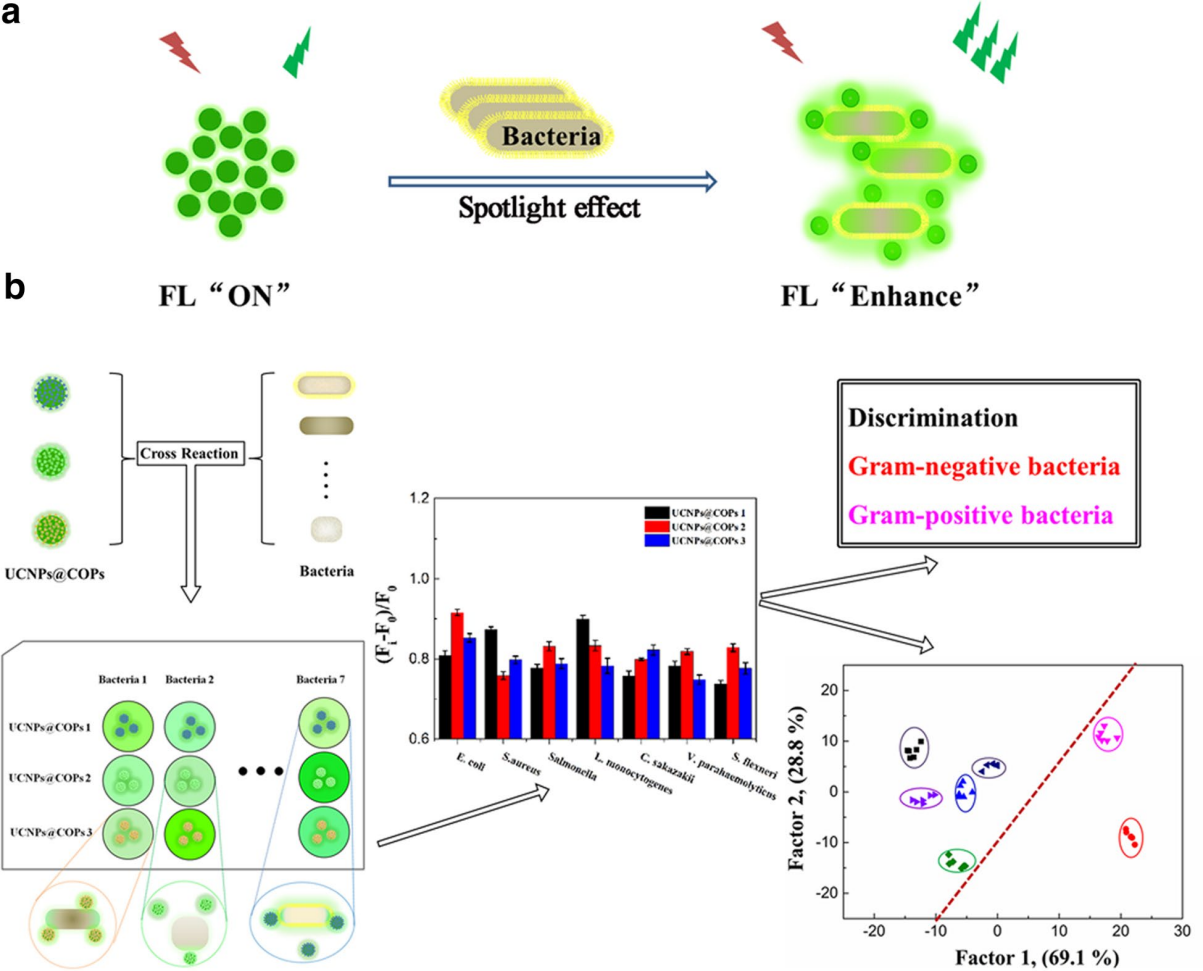
Schematic illustration of pattern recognition of bacteria based on UCNPs@COPs materials fluorescence sensor array. **a** Principle of fluorescence emission intensity enhancement of UCNPs materials. **b** The designed functional UCNPs@COPs made cross reaction with bacteria to construct the sensors array, and the obtained fluorescent response patterns was utilized to identify and classify bacteria through linear discriminant analysis (LDA)

Generally, the bacterial cell can improve the fluorescence emission of UCNPs material due to the spotlight effect of cell and the interaction [10–12]. The three UCNPs@COPs probes with different functional groups can further reinforce the interaction and exhibit the different fluorescence signal response. The strongly positive charged imidazole ionic liquid groups and negative charged phosphate groups can interact with the charged groups on the surface of bacteria. Phenylboronic acid can interact with the peptidoglycan and teichoic acid on the surface of bacteria. Certain surface groups and charge property of each kind of bacteria may lead to different binding intensity. Thus, we expected that the common foodborne pathogenic bacteria with phenotypes differences could be reflected from the fluorescence signal. Furthermore, each UCNPs@COPs probe could respond to the various species of bacteria in a unique manner and generate corresponding features fluorescence signal. The three UCNPs@COPs probes could be employed to constitute a three-probe sensor array to realize the identification of bacterial species (Scheme 1).

In an attempt to optimize the working conditions of the three UCNPs@COPs probes, the ubiquitous and well-studied Gram-negative *E.coli* was chosen as a model bacterium. The physiological saline containing 10% DMSO was selected as the working media, which could well maintain the dispersion of UCNPs probes and didn’t damage the bacterial cell morphology. The effect of the concentrations of UCNPs@COPs probes and incubation time against *E.coli* were first examined. As shown in Additional file 1: Figures S8 and S9, the relative fluorescence intensities ((F_i_ − F_0_)/F_0_) of the three UCNPs@COPs probes towards bacteria varied with the increase of UCNPs@COPs concentrations and incubation time. Consistently, the fluorescence emission intensities of the three UCNPs@COPs probes could be enhanced by *E.coli* (OD_600_ = 0.5). The strongest fluorescence response was obtained after incubation about 6 min at 0.20 mg mL^−1^ of each UCNPs@COPs probe. Therefore, such probe concentration and incubation time were adopted for the following experiments. Meanwhile, the bacterial concentration played an important role. As shown in the Additional file 1: Figure S10, the fluorescence emission of each fluorescence probe enhanced with the increase of *E.coli* concentration, and the difference among these emissions was the largest at OD_600_ = 0.5. Such bacterial concentration (OD_600_ = 0.5) was preliminary chosen for the following experiments.

**Fig. 3:**
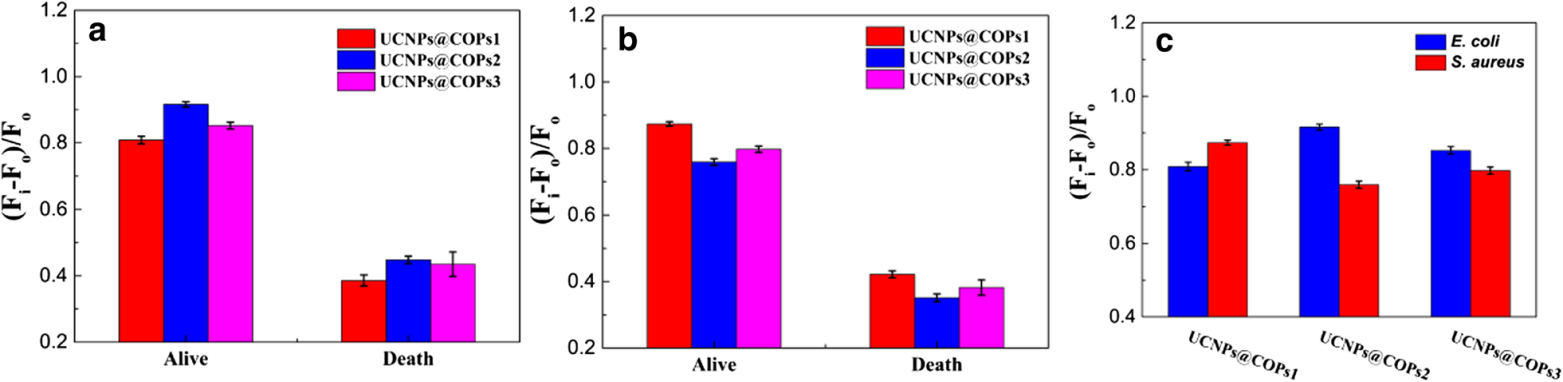
**a** Fluorescent response of UCNPs@COPs probes treated with alive and death *E. coli* (OD_600_ = 0.5). **b** Fluorescent response of UCNPs@COPs probes treated with alive and death *S. aureus* (OD_600_ = 0.5). **c** Fluorescent response of UCNPs@COPs probes treated with alive *E. coli* and *S. aureus* (OD_600_ = 0.5)

In order to further evaluate the discrimination ability of UCNPs@COPs probes, each UCNPs@COPs probe was treated with alive or dead *E.coli* and *S. aureus*, respectively, and their fluorescence responses were recorded. As we expected, the fluorescence probes could realize the identification of the alive or dead *E.coli* and *S. aureus*. The alive *E.coli* and *S. aureus* could enhance the fluorescence of UCNPs@COPs probes more significant than that of the dead one treated with ultrasonic (Fig. 3a, b).

Furthermore, the fluorescence responses of the three UCNPs@COPs probes towards Gram-negative *E. coli* and Gram-positive *S. aureus* were preliminary investigated. As seen in Fig. 3c, the fluorescence responses of each UCNPs@COPs probe towards *E. coli* and *S. aureus* were obvious. Each UCNPs@COPs probe showed different fluorescence responses towards the two bacteria, which was mainly on account of the difference of morphology between *E. coli* (thallus with blunt ends) and *S. aureus* (small spherical thallus). Meanwhile, all the three UCNPs@COPs probes revealed unique fluorescence response against each kind of bacterium, and the dramatic enhancement could reflect the fairly degree of interaction between UCNPs@COPs probes and bacteria due to the differences in binding ability. Obviously, UCNPs@COPs 1 (UCNPs modified with boronic acid groups) displayed the strongest fluorescence emission towards *S. aureus* than that of UCNPs@COPs 2 (UCNPs modified with imidazole ionic liquid). The reasons were attributed to the facts that there were more peptidoglycan and teichoic acid on the surface of Gram-positive bacteria, and boronic acid groups combine strongly with cis-diol molecules. On the other hand, more negative surface charges on the surface of Gram-negative bacteria led to the highest fluorescence emission of UCNPs@COPs 2 towards *E. coli* due to the strong electrostatic interaction between imidazole quaternary ammonium salt and negative charge groups on the surface of *E. coli*. In addition, UCNPs@COPs 3 (UCNPs modified with negative charge phosphate groups) could form weak electronic interaction with the molecules on bacterial cell walls and generate the fluorescence response, however, which was the weakest among these probes. Herein, considering the complexity of bacteria, we concluded that both the spotlight effect of bacterial cell and the interaction between bacteria and the fluorescence probes were the possible sensing mechanisms. The extents of fluorescence response of UCNPs@COPs probes were dependent on the types of bacteria and their multiple surface molecules, which could provide the possibility to distinguish diverse bacteria.

### Bacteria detection and discrimination using fluorescent sensor array

**Fig. 4:**
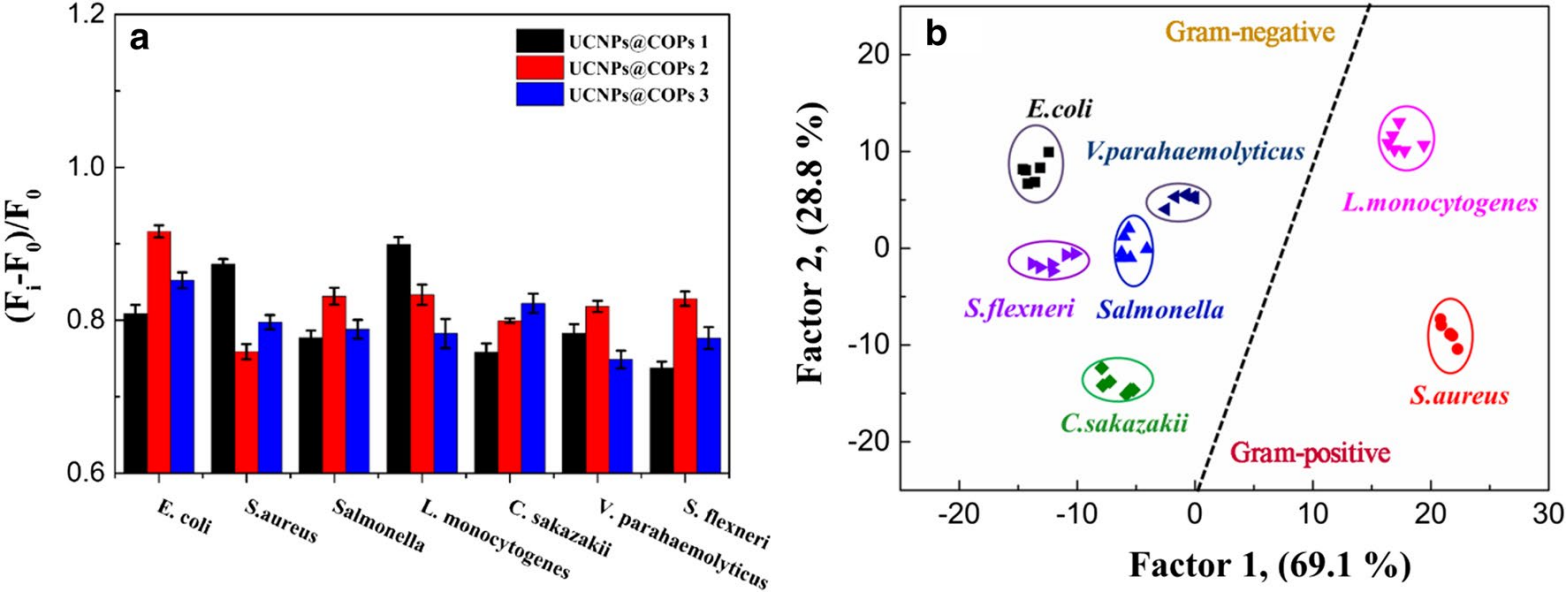
**a** Signal patterns of the relative fluorescence intensity variety ((F_i_ − F_0_)/F_0_) of three kinds of UCNPs@COPs probes in the presence of seven different bacteria (OD_600_ = 0.5). All values represent the average of six replicates. **b** Canonical score plot for the response patterns of bacteria obtained from LDA. Each point represents the response pattern for single bacterial species to the array

Since UCNPs@COPs probes could generate the different fluorescent response towards diverse bacteria, we further investigated the fluorescent performances of UCNPs@COPs probes towards seven kinds of common foodborne pathogenic bacteria (including *E. coli*, *S. aureus*, *Salmonella*, *L. monocytogenes*, *C. sakazakii*, *S. flexneri*, and *V. parahaemolyticus*). To avoid unnecessary signal fluctuation, the seven kinds of bacteria with a concentration (OD_600_ = 0.5) were incubated with 0.20 mg mL^−1^ of three UCNPs@COPs probes in physiological saline containing 10% DMSO for 6 min, and the physiological saline alone as a control. All tests were performed in six replicates. Then the fluorescence spectra were recorded, and the parameter [(F_i_ − F_0_)/F_0_] was further utilized to characterize the fluorescence response patterns of each UCNPs@COPs-based materials against the seven kinds of bacteria. To our delight, the three probes exhibited significant fluorescence response towards each kind of bacterium (Fig. 4a). The fluorescence response was closely related to the bacterial species, and the achieved fluorescence response could be utilized as the “identification fingerprints” of bacteria for the specific discrimination.

To further maximize the separation ability of the fluorescent sensor array, the fluorescence response patterns of the sensor array were combined, producing a training matrix (3 × UCNPs@COPs × 7 bacteria × 6 replicates). Then the matrix was classified by linear discriminant analysis (LDA) using the analysis software SPSS (version 22.0), which was a powerful statistical method to separate and identify multiple targets simultaneously by using their linear combination of features. LDA converted the training matrix into canonical scores according to their Mahalanobis distance (Additional file 1: Table S3). Three canonical factors (69.1%, 28.8%, and 2.1%) were generated for the total variance. The 2D canonical scores were plotted according to the first two most important discrimination factors where each point represented the fluorescence response of UCNPs@COPs to each kind of bacteria. We observed that the seven kinds of bacteria were well-clustered without any overlap and discriminated thoroughly from each other (Fig. 4b). The 100% classification accuracy for each set of bacteria was confirmed according to Jackknife classification (Additional file 1: Table S4), suggesting the extraordinary properties of the three-channel sensor array in discrimination of bacteria. Seven bacteria clustered independently according to their categories in the 2D canonical scores plot, where the Gram-positive bacteria positioned at the right and the Gram-negative bacteria at the left, indicating a certain correlation between bacterial Gram types and the three UCNPs@COPs probes. Furthermore, we simplified the sensor array by removal of one UCNPs@COPs probe. However, the satisfactory discrimination between seven bacteria was not achieved based on the two-channel UCNPs@COPs sensor array (Additional file 1: Figure S11), indicating that more different UCNPs@COPs probes could distinguish more bacteria.

**Fig. 5:**
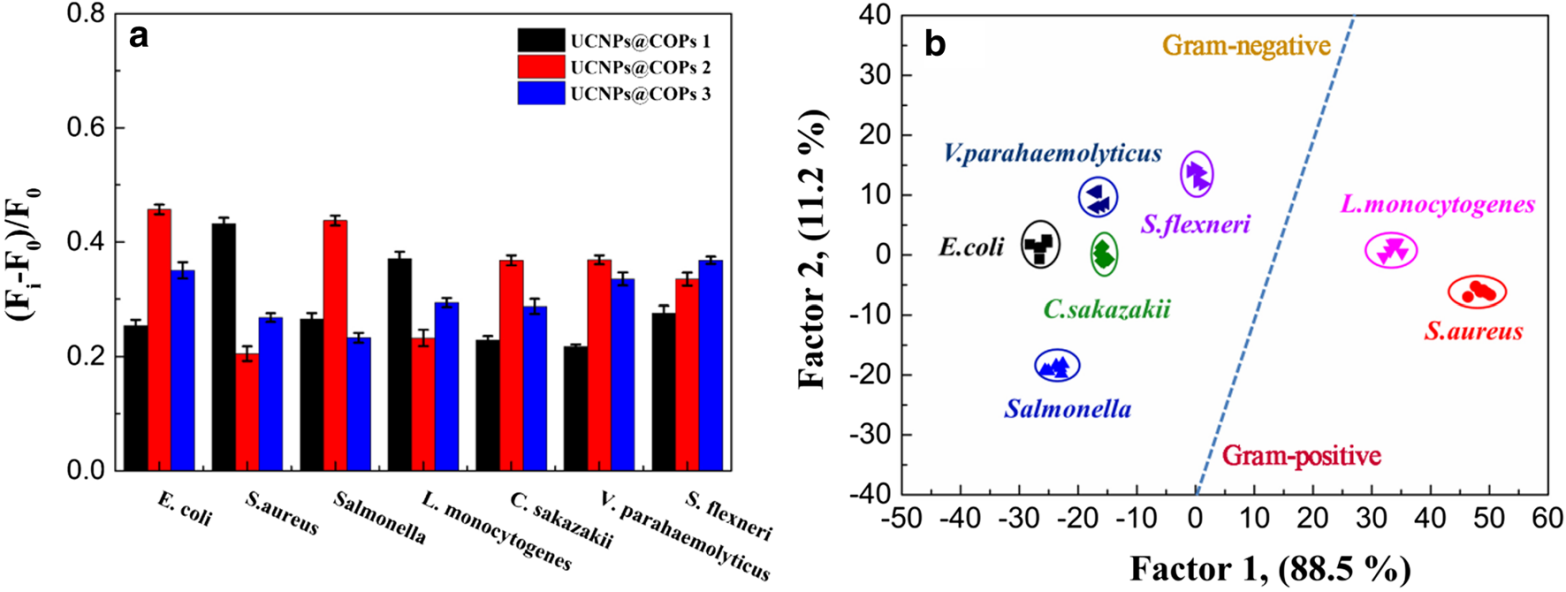
**a** Signal patterns of the relative fluorescence intensity variety ((F_i_ − F_0_)/F_0_) of three kinds of UCNPs@COPs probes in the presence of seven different bacteria (OD_600_ = 0.1). All values represent the average of six replicates. **b** Canonical score plot for the response patterns of bacteria obtained from LDA. Each point represents the response pattern for single bacteria species to the array

To validate the efficiency of the fluorescence sensor array, we further decreased the concentration of bacteria to OD_600_ = 0.1 (approximates to 10^6^ cfu mL^−1^ seen in Additional file 1: Table S2). To our delight, the fluorescent sensor array still revealed a good discrimination and accuracy towards the low concentration bacteria (Fig. 5 and Additional file 1: Table S5). Therefore, our fluorescent sensor array displayed better recognition ability and a higher sensitivity (OD_600_ = 0.1) over the previous report (OD_600_ = 1.0) [3, 18].

**Fig. 6:**
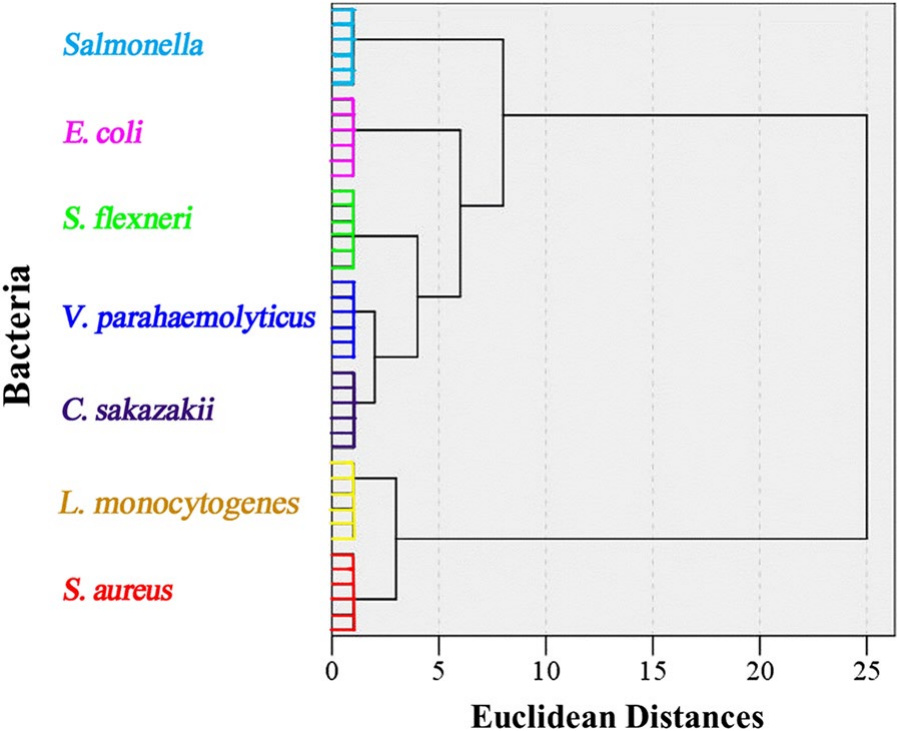
Dendrogram generated by hierarchical cluster analysis of the seven different bacteria

In order to further explore the ability of this fluorescent sensor array, HCA was employed to analyse the similarities between the selected bacteria in a step-by-step way according to the Euclidean distances, which was a powerful classify method [18]. The dendrogram produced from HCA based on average linkage method obviously revealed seven distinct clusters, and each cluster represented one kind of bacteria (Fig. 6). Meanwhile, the four Gram-negative bacteria (*E. coli*, *Salmonella*, *C. sakazakii*, *V. parahaemolyticus*, and *S. flexneri*) matched at the same level, whereas the two Gram-negative bacteria (*S. aureus* and *L. monocytogenes*) at the same level, revealing the more similarity among the bacteria. The results demonstrated that the fluorescent sensor array could discriminate the different Gram states of bacteria.

### Discrimination of bacteria mixtures

**Fig. 7:**
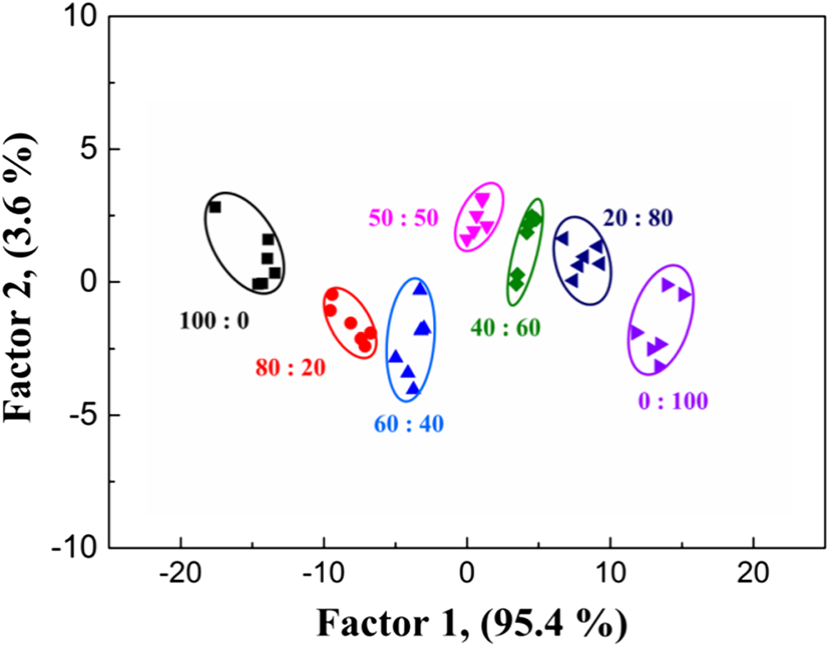
Canonical score plot for the UCNPs@COPs-based fluorescent sensor array against the bacteria mixtures of *E. coli* and *S. aureus* with different ratios (100:0, 80:20, 60:40, 50:50, 40:60, 20:80, and 0:100), respectively in physiological saline. In each case, a bacteria concentration of OD_600_ = 0.1 was used. Each point represents the response pattern for single bacterial mixtures to the array

The coexisting of foodborne bacteria in the nature environment was not fresh, which make it of great significance to distinguish the bacteria mixture. In order to investigate the performance of this fluorescent sensor array towards bacteria mixture, *E. coli* and *S. aureus* were employed as the model, and the mixtures of two model bacteria with different ratios (100:0, 80:20, 60:40, 50:50, 40:60, 20:80, and 0:100) were design to simulated bacterial coexistence. Then, each mixture with the concentration of OD_600_ = 0.1 was identified using the constructed upconversion fluorescent sensor array (Additional file 1: Table S6). The resultant canonical score plot has shown that the different ratios of bacterial mixtures could be successfully separated without any overlapping, which further proved the potential ability of the proposed fluorescent sensor array for discriminating bacteria mixture (Fig. 7).

### Quantitative analysis of bacteria

**Fig. 8:**
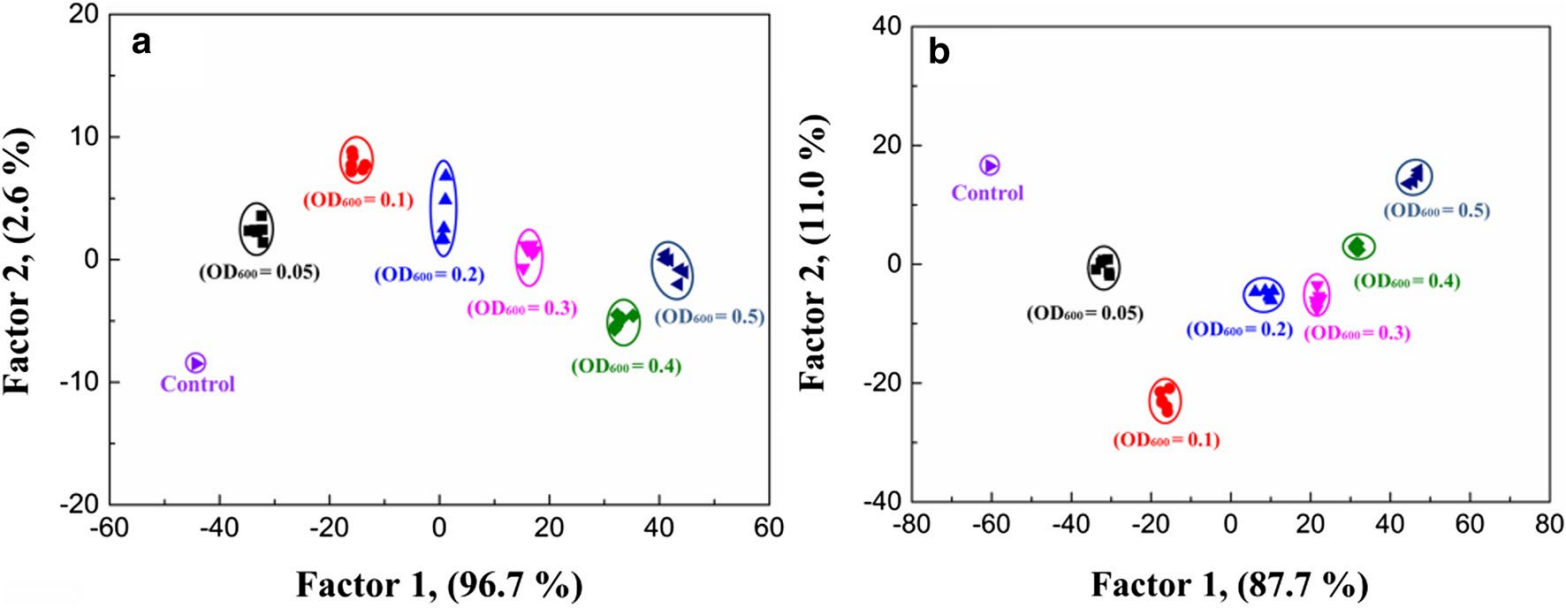
2D canonical score plots obtained with the fluorescent sensor array treated with *E.coli* (**a**) and *S. aureus* (**b**) at different concentrations (OD_600_ from 0.5 to 0.05). Each point represents the response pattern for single bacterial concentrations to the array

Since the fluorescence intensities of UCNPs@COPs probes increased with the concentration of bacteria, *E.coli* and *S. aureus* were selected to test the quantitative analysis performance of the proposed fluorescent sensor array by using LDA (6 concentrations × 3 UCNPs@COPs × 6 replicates, OD_600_ from 0.05 to 0.50, Additional file 1: Tables S7 and S8). The result showed that all of the six concentrations of *E.coli* or *S. aureus* could be discriminated with 100% accuracy without any overlap, indicating that the proposed fluorescent sensor array could be well employed for the quantitative analysis of bacteria (Fig. 8).

### Identification of bacteria in real samples

**Fig. 9:**
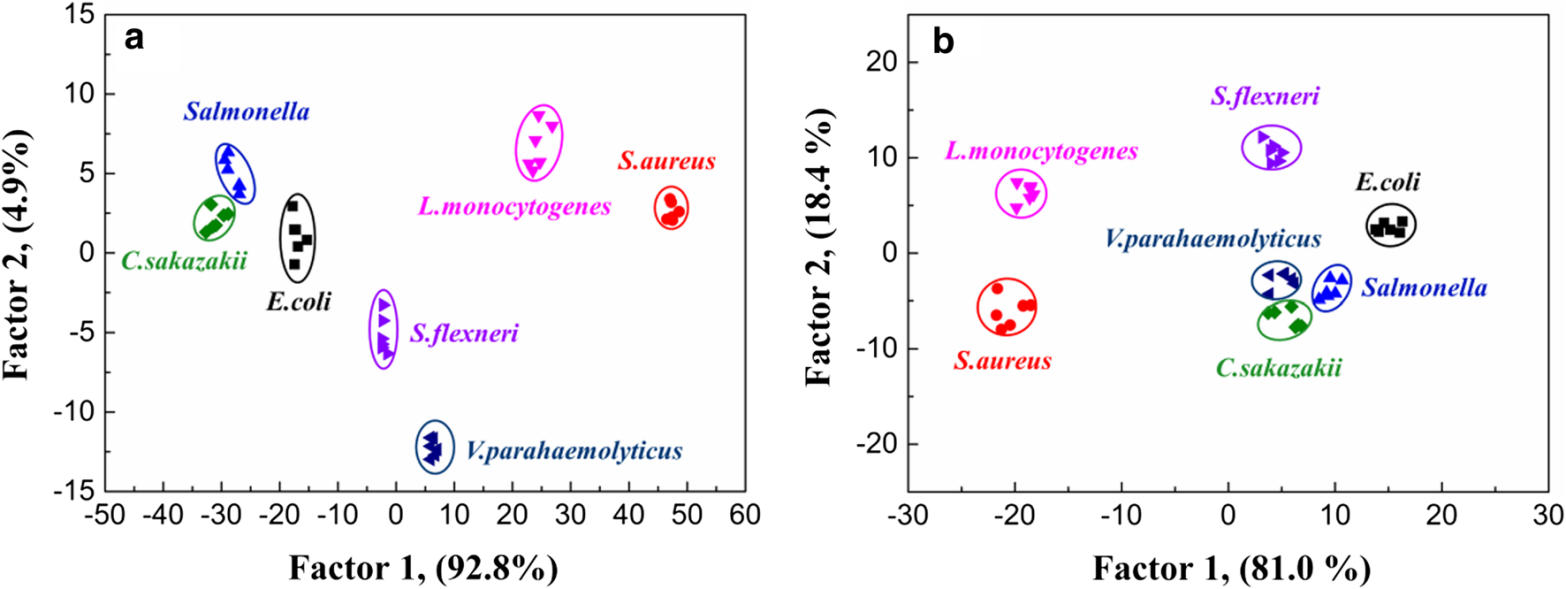
Discrimination of bacteria in milk and beef culture solution. **a** LDA plot for the discrimination of bacteria in milk (OD_600_ = 0.1). **b** LDA plot for the discrimination of bacteria in beef culture solution (OD_600_ = 0.1). Each point represents the response pattern for single bacterial species to the array

In order to verify the performance of the fluorescent sensor array in real food samples, we further simulated bacterial contaminated food (milk and ground beef) to test the discrimination capacity. The proposed fluorescent sensor array was employed to discriminate the common seven foodborne bacteria in these food samples (3 × UCNPs@COPs × 7 bacteria × 6 replicates). The training results in presence of the food samples matrix interference (milk and beef) showed that the bacteria could be successfully discriminated in complex samples with the same high-accuracy as in physiological saline, indicating that the sensor array equipped with a good capacity of resisting disturbance (Fig. 9, Additional file 1: Tables S9 and S10). Furthermore, 63 unknown bacteria were spiked in tap water, milk and beef culture solution respectively with a bacteria concentration (about 10^6^ cfu mL^−1^), which were randomly picked from the seven bacteria. Detection and identification of unknown bacteria samples were carried out by assigning the fluorescence response pattern of the unknown samples to the corresponding LDA group defined by the training matrix (Additional file 1: Tables S11–S13). The blind tests results showed 92.1% accuracy of bacterial discrimination and the results were reliable in tap water with 100% accuracy of discrimination, allowing for higher accuracy in discrimination of bacteria in beef (90.5%) than that in milk (85.7%). The results indicated that the proposed fluorescent sensor array based on UCNPs@COPs materials is great potential for practical applications (Table 1). In addition, the discrimination of bacteria mixtures (*E. coli* and *S. aureus*) with different ratios in real samples was further investigated. The resultant canonical score plot could be successfully separated without any overlapping, which further demonstrated the excellent discriminating capability of the proposed fluorescent sensor array for bacteria mixture in real samples (Additional file 1: Figure S12 and Tables S14–S16).

**Table 1:** Accuracy (%) of the blind tests obtained from the fluorescent sensor array

Bacteria source	Tap water	Milk	Beef	Total
Number of samples	21	21	21	63
Correctly identified	21	18	19	58
Accuracy (%)	100%	85.7%	90.5%	92.1%

## Conclusion

A novel upconversion fluorescent sensor array was constructed for discrimination of bacteria based on surface chemistry modified UCNPs materials with boronic acid, quaternary ammonium salt or phosphate groups. The sensing mechanism was mainly depended on the spotlight effect of bacterial cell and interactions between bacteria and the fluorescent probes. The proposed fluorescent sensor array allowed for the quantitative analysis of bacteria and the good discrimination and classification towards bacteria according to the Gram status. In comparison to previous pattern based microbial identification, intact alive microbes instead of microbial lysates were straightly chosen as analytical target, which could shorten preprocessing and realize fast detection and real-time monitoring (Additional file 1: Table S17). Excellent discriminating power in bacteria mixture and real samples (tap water, milk and beef) encouraged us that the sensor array might accelerate the development of the efficient discrimination of complex bacterial samples.

## Supplementary information

**Additional file 1.** Additional figures and tables.

## Data Availability

All data generated or analyzed during this study are included in the article and additional file. The additional file is available. Synthesis process of UCNPs, IL-Br, and Co-polymers. EDS, XRD patterns, FITR spectrum, Thermogravimetric analysis, Zeta potential, and Fluorescence emission spectra of UCNPs@COPs materials (Additional file 1: Figures S1–S7). Optimization of assay conditions of the fluorescent sensor array (Additional file 1: Figures S8–S11). Linear discriminant analysis using the sensor array against the bacteria mixtures in real samples (Figure S11). Synthesis of co-polymers (Additional file 1: Table S1). Numbers of bacteria using plate count (Additional file 1: Table S2). Fluorescence response pattern and linear discriminant analysis (Additional file 1: Tables S3–S10 and S14–S16) Detection and identification of unknown bacteria samples (Additional file 1: Tables S11–S13). Comparison of different detection methods (Additional file 1: Table S17).

## Abbreviations

UCNPs: Upconversion nanoparticles
COPs: Co-polymers
TEOS: Tetraethyl orthosilicate
ATS: Allyltriethoxysilane
AIBN: Azodiisobutyronitrile
DMSO: Dimethyl sulfoxide
PBS: Phosphate buffer saline
TEM: Transmission electron microscopy
EDS: Energy-dispersive X-ray spectroscope
FT-IR: Fourier transform infrared spectra
TGA: Thermogravimetric analysis
LDA: Linear discriminant analysis
