# Supplementary material for: Surface chemistry modified upconversion nanoparticles as fluorescent sensor array for discrimination of foodborne pathogenic bacteria
Source: J Nanobiotechnology. 2020 Feb 28;18:41. doi: 10.1186/s12951-020-00596-4 (PMC7049179; doi:10.1186/s12951-020-00596-4)
Supplement: Supplementary file 1 — Additional file 1. Additional figures and tables. [file 12951_2020_596_MOESM1_ESM.docx]

**Surface chemistry Modified Upconversion Nanoparticles as Fluorescent Sensor Array for Discrimination of Foodborne Pathogenic Bacteria**

Mingyuan Yin^1^, Chuang Jing^1^, Haijie Li^1^, Qiliang Deng*^,1^, Shuo Wang*^,1,2^

^1^ State Key Laboratory of Food Nutrition and Safety, School of Food Engineering and Biotechnology, College of Chemical Engineering and Materials Science, Tianjin University of Science and Technology, Tianjin 300457, People’s Republic of China.

^2^ Tianjin Key Laboratory of Food Science and Health, School of Medicine, Nankai University, Tianjin, 300071, People’s Republic of China.

* Corresponding author: Qiliang Deng or Shuo Wang. E-mail: [yhdql@tust.edu.cn](mailto:yhdql@tust.edu.cn) or wangshuo@nankai.edu.cn

**Synthesis of UCNPs**

The oil-solvent UCNPs were prepared as previous procedure.[[1](#_ENREF_1)] In a typical experiment, 1mM RE (CH_3_COO)_3_ [Y:Yb:Er 78:20:2] was dissolved in a three-necked flask with OA (6 mL) and ODE (17 mL), and this system was kept at 160 °C for 30 min to form a transparent solution under argon atmosphere and vigorous stirring. Next, the system was kept at room temperature for 1h, and 10 mL methanol solution with NaOH (2.5 mM) and NH_4_F (4 mM) was added to keep for another 30 min. Then methanol was removed by heated evaporation at 70 °C. Subsequently, the system was kept at 300 °C for 1 h with argon atmosphere and vigorous stirring, then cooled down to collect UCNPs via centrifugation (12186 × g, 10 min). The obtained materials were washed with ethanol and dried in air.

**Synthesis of** **1-octyl-3-vinylimidazolium bromide ionic liquid (IL-Br)**

IL-Br was synthesized according to the protocol from the literature.[[2](#_ENREF_2)] A mixture solution containing 1-bromoctane monomer (0.075 mol) and 1-vinylimidazole (0.075 mol) was stirred at room temperature for 24 h. The obtained product was washed with ethyl acetate and diethyl ether three times respectively, and then dried under dynamic vacuum at room temperature for 24 h.

**Synthesis of Co-polymers**

The co-polymers (COPs) were obtained by the free radical polymerization reaction, and the protocol was slightly modified the previous procedure. [[3](#_ENREF_3)] The allyltriethoxysilane (ATS) and the functional vinyl monomers (VPBA, IL-Br and VPA) were mixed respectively and initiated to form COPs (ATS-co-ligands) by azodiisobutyronitrile (AIBN) in acetonitrile (6.0 ml) at 60 °C for 12 h under stirring (Table S1).


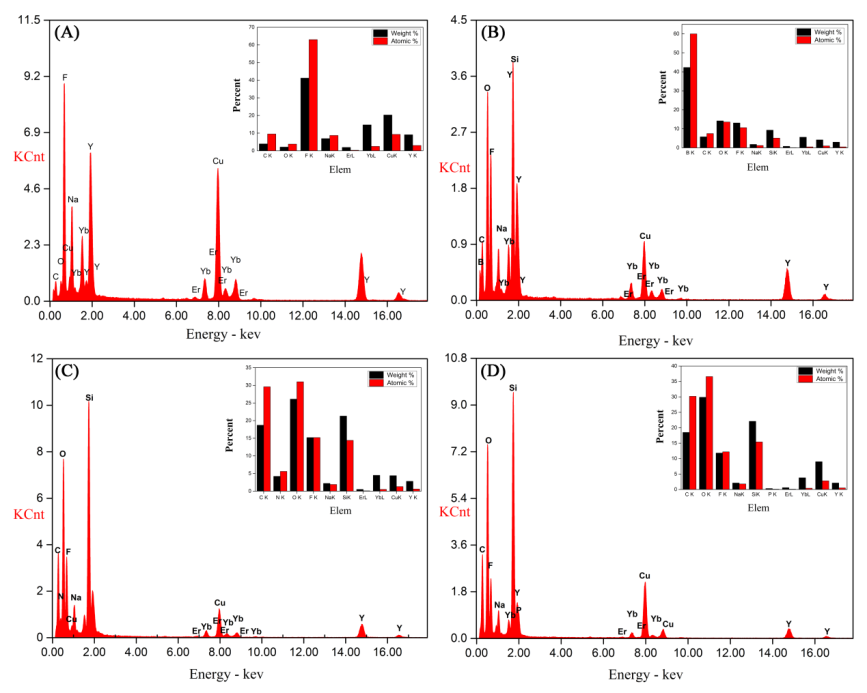


**Figure S1** EDS of UCNPs (A), UCNPs@COPs 1 (B), UCNPs@COPs 2 (C), and UCNPs@COPs 3 (D).





**Figure S2** XRD patterns of UCNPs (a), UCNPs@COPs 1 (b), UCNPs@COPs 2 (c), UCNPs@COPs 3 (d), and standard alignment card (s).





**Figure S3** FI-TR spectrum of UCNPs@COPs materials.

*
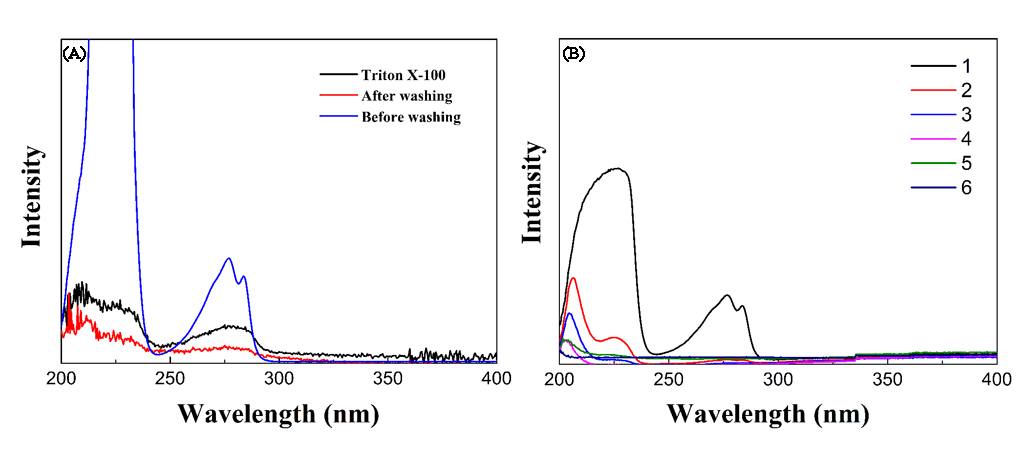
*

**Figure S4** (A) UV-Vis absorbance spectra of the pre-treated and post-treated UCNPs@COPs materials. (B) The change of UV-Vis absorbance spectra of ethanol washing solution with the washing times.





**Figure S5** Thermogravimetric analysis of UCNPs@COPs materials.





**Figure S6** Zeta potential of UCNPs@COPs materials.


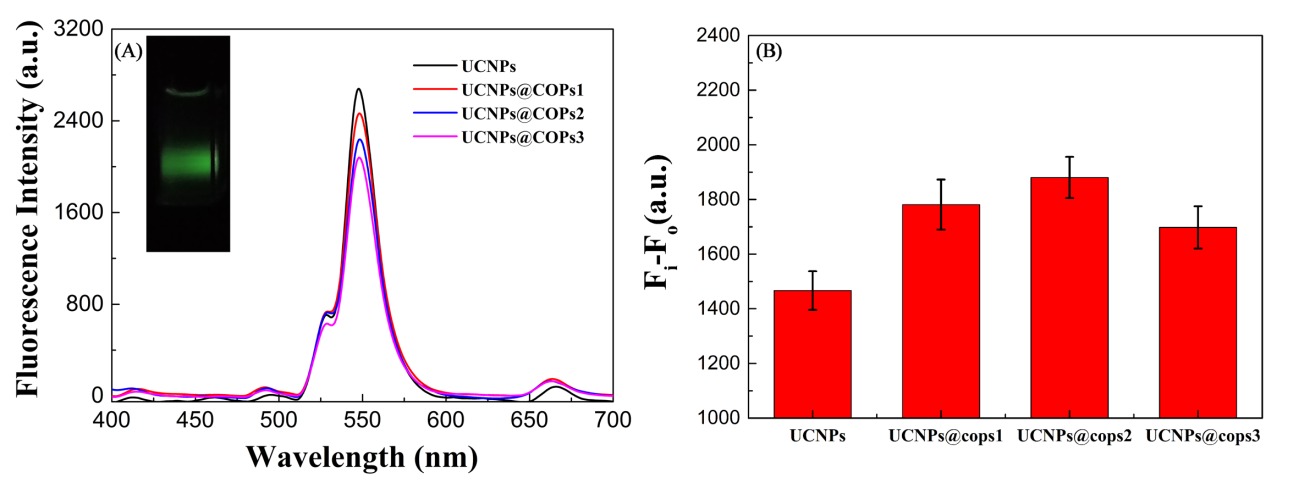


**Figure S7** (A) Fluorescence emission spectra of UCNPs materials. (B) Fluorescent response of UCNPs materials treated with alive E. coli (OD_600_ = 0.50).


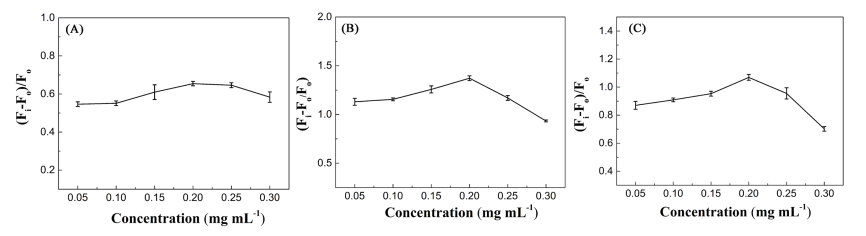


**Figure S8** Optimization of assay conditions for the concentration of UCNPs@COPs probes treated with the model *E.coli* (OD_600_=0.5). (A) UCNPs@COPs 1, (B) UCNPs@COPs 2, and (C) UCNPs@COPs 3.


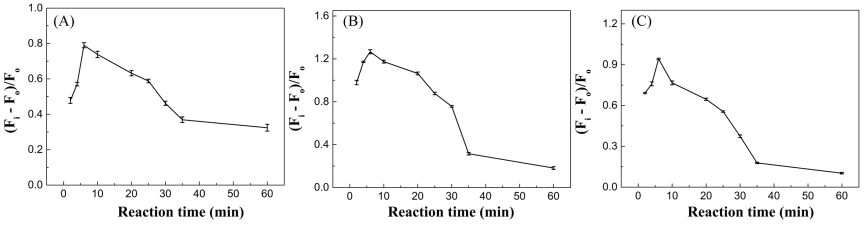


**Figure S9** Optimization of assay conditions for the incubation time of UCNPs@COPs probes treated with the model *E.coli* (OD_600_=0.5). (A) UCNPs@COPs 1, (B) UCNPs@COPs 2, and (C) UCNPs@COPs 3.





**Figure S10** Relative fluorescence intensity variety ((F_i_-F_0_)/F_0_) of UCNPs@COPs probes towards different concentration of *E.coli* (OD_600_ = 0.05, 0.1, 0.2, 0.3, 0.4, and 0.5).


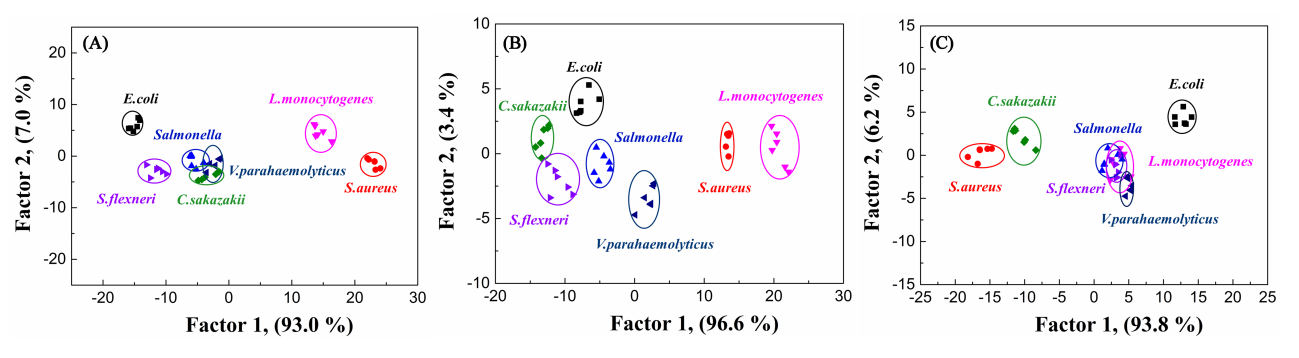


**Figure S11** Canonical score plot for bacteria discrimination based on sensor array of (A) UCNPs@COPs 1 + UCNPs@COPs 2, (B) UCNPs@COPs 1 + UCNPs@COPs 3, and (C) UCNPs@COPs 2 + UCNPs@COPs 3.


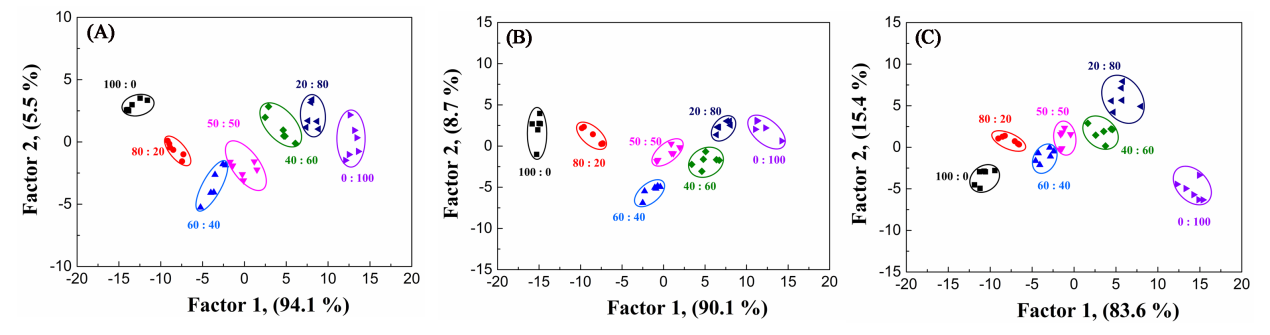


**Figure S12** Canonical score plot for the UCNPs@COPs-based fluorescent sensor array against the bacteria mixtures of *E. coli* and *S. aureus* with different ratios (100:0, 80:20, 60:40, 50:50, 40:60, 20:80, and 0:100), respectively from (A) tap water, (B) milk and (C) beef. In each case, a bacteria concentration (about 10^6^ cfu mL^-1^) was used. Each point represents the response pattern for single bacterial mixtures to the array.

**Table S1** Synthesis of co-polymers.

| Name | Co-polymer | Ligand 1  (0.04 mM) | Ligand 2  (0.04 mM) | Initiator  (3 %) |
| --- | --- | --- | --- | --- |
| COPs 1 | poly (ATS-co- VPBA) | VPBA | ATS | AIBN |
| COPs 2 | poly (ATS-co- IL-Br) | IL-Br |  |  |
| COPs 3 | poly (ATS-co-VPA) | VPA |  |  |

**Table S2** Numbers of bacteria (cfu mL^-1^) at OD_600_=0.1 using plate count.

| Nr | Name of Bacteria | Gram | OD_600_ | Corresponding Numbers of Bacteria (cfu mL^-1^) |
| --- | --- | --- | --- | --- |
| 1 | *E. coli* | negative | 0.1 | 10^6^ |
| 2 | *Salmonella* | negative | 0.1 | 10^6^ |
| 3 | *C. sakazakii* | negative | 0.1 | 10^6^ |
| 4 | *S. flexneri* | negative | 0.1 | 10^6^ |
| 5 | *V. parahaemolyticus* | negative | 0.1 | 10^6^ |
| 6 | *S. aureus* | positive | 0.1 | 10^6^ |
| 7 | *L. monocytogenes* | positive | 0.1 | 10^6^ |

**Fluorescence Response Pattern and Linear Discriminant Analysis**

**Table S3.** Training matrix of fluorescence response pattern obtained from an array of UCNPs@COPs probes against 7 bacteria (OD_600_=0.5) in physiological saline. LDA was carried out and resulting in 3 factors of the canonical scores and group generation. Jackknifed classification matrix showed the 100% correct classification.

| Analytes  Bacteria | Fluorescence Response Pattern | | | Results LDA | | | Group |
| --- | --- | --- | --- | --- | --- | --- | --- |
|  | C1 | C2 | C3 | F1 | F2 | F3 |  |
| *E. coli* | 0.824 | 0.928 | 0.870 | -13.127 | 8.295 | 5.415 | 1 |
| *E. coli* | 0.821 | 0.923 | 0.855 | -12.405 | 9.923 | 4.331 | 1 |
| *E. coli* | 0.808 | 0.913 | 0.855 | -13.578 | 6.821 | 4.126 | 1 |
| *E. coli* | 0.801 | 0.913 | 0.845 | -14.345 | 8.047 | 3.423 | 1 |
| *E. coli* | 0.800 | 0.913 | 0.844 | -14.604 | 8.160 | 3.312 | 1 |
| *E. coli* | 0.797 | 0.906 | 0.844 | -14.183 | 6.665 | 3.232 | 1 |
| *S. aureus* | 0.879 | 0.770 | 0.804 | 20.817 | -7.336 | 1.436 | 2 |
| *S. aureus* | 0.877 | 0.767 | 0.804 | 20.890 | -7.973 | 1.375 | 2 |
| *S. aureus* | 0.877 | 0.762 | 0.803 | 21.624 | -8.880 | 1.301 | 2 |
| *S. aureus* | 0.877 | 0.761 | 0.802 | 21.649 | -8.841 | 1.234 | 2 |
| *S. aureus* | 0.868 | 0.747 | 0.791 | 22.264 | -10.425 | 0.377 | 2 |
| *S. aureus* | 0.863 | 0.747 | 0.781 | 21.832 | -9.049 | -0.363 | 2 |
| *Salmonella* | 0.785 | 0.842 | 0.806 | -6.236 | -0.461 | 0.493 | 3 |
| *Salmonella* | 0.784 | 0.842 | 0.797 | -6.031 | 1.229 | -0.165 | 3 |
| *Salmonella* | 0.783 | 0.840 | 0.790 | -5.624 | 2.056 | -0.639 | 3 |
| *Salmonella* | 0.779 | 0.825 | 0.783 | -4.078 | -0.070 | -1.187 | 3 |
| *Salmonella* | 0.767 | 0.824 | 0.781 | -6.261 | -0.991 | -1.453 | 3 |
| *Salmonella* | 0.763 | 0.817 | 0.772 | -5.488 | -0.976 | -2.122 | 3 |
| *L. monocytogenes* | 0.911 | 0.849 | 0.806 | 16.401 | 10.846 | 2.200 | 4 |
| *L. monocytogenes* | 0.909 | 0.846 | 0.798 | 16.772 | 11.623 | 1.607 | 4 |
| *L. monocytogenes* | 0.903 | 0.833 | 0.789 | 17.775 | 10.089 | 0.942 | 4 |
| *L. monocytogenes* | 0.894 | 0.830 | 0.782 | 16.927 | 10.196 | 0.329 | 4 |
| *L. monocytogenes* | 0.891 | 0.829 | 0.763 | 17.320 | 13.008 | -0.931 | 4 |
| *L. monocytogenes* | 0.889 | 0.813 | 0.758 | 19.410 | 10.661 | -1.348 | 4 |
| *C. sakazakii* | 0.771 | 0.803 | 0.835 | -5.231 | -14.635 | 2.062 | 5 |
| *C. sakazakii* | 0.768 | 0.802 | 0.832 | -5.467 | -14.620 | 1.828 | 5 |
| *C. sakazakii* | 0.765 | 0.800 | 0.831 | -5.825 | -15.094 | 1.713 | 5 |
| *C. sakazakii* | 0.753 | 0.799 | 0.818 | -7.221 | -13.773 | 0.703 | 5 |
| *C. sakazakii* | 0.748 | 0.796 | 0.815 | -7.806 | -14.187 | 0.395 | 5 |
| *C. sakazakii* | 0.744 | 0.796 | 0.802 | -7.919 | -12.370 | -0.451 | 5 |
| *V. parahaemolyticus* | 0.797 | 0.826 | 0.763 | -0.002 | 5.208 | -2.232 | 6 |
| *V. parahaemolyticus* | 0.794 | 0.825 | 0.761 | -0.230 | 5.272 | -2.423 | 6 |
| *V. parahaemolyticus* | 0.781 | 0.821 | 0.750 | -1.687 | 5.305 | -3.335 | 6 |
| *V. parahaemolyticus* | 0.781 | 0.816 | 0.743 | -0.728 | 5.521 | -3.783 | 6 |
| *V. parahaemolyticus* | 0.780 | 0.815 | 0.742 | -0.807 | 5.529 | -3.860 | 6 |
| *V. parahaemolyticus* | 0.763 | 0.807 | 0.733 | -2.487 | 4.033 | -4.672 | 6 |
| *S. flexneri* | 0.743 | 0.843 | 0.796 | -13.817 | -1.581 | -0.764 | 7 |
| *S. flexneri* | 0.743 | 0.832 | 0.788 | -12.081 | -2.346 | -1.285 | 7 |
| *S. flexneri* | 0.740 | 0.831 | 0.782 | -12.148 | -1.659 | -1.762 | 7 |
| *S. flexneri* | 0.740 | 0.825 | 0.770 | -10.935 | -0.702 | -2.556 | 7 |
| *S. flexneri* | 0.738 | 0.819 | 0.762 | -10.182 | -0.558 | -3.123 | 7 |
| *S. flexneri* | 0.721 | 0.818 | 0.761 | -13.150 | -1.960 | -3.382 | 7 |

**Table S4.** Summary of the jackknifed classification matrix for seven kinds of bacteria.

|  | *E. coli* | *S. aureus* | *Salmonella* | *L. monocytogenes* | | *C. sakazakii* | | *S. flexneri* | *V.parahaemolyticus* | %  correct |
| --- | --- | --- | --- | --- | --- | --- | --- | --- | --- | --- |
| *E. coli* | 6 | 0 | 0 | 0 | 0 | | 0 | | 0 | 100 |
| *S. aureus* | 0 | 6 | 0 | 0 | 0 | | 0 | | 0 | 100 |
| *Salmonella* | 0 | 0 | 6 | 0 | 0 | | 0 | | 0 | 100 |
| *L. monocytogenes* | 0 | 0 | 0 | 6 | 0 | | 0 | | 0 | 100 |
| *C. sakazakii* | 0 | 0 | 0 | 0 | 6 | | 0 | | 0 | 100 |
| *S. flexneri* | 0 | 0 | 0 | 0 | 0 | | 6 | | 0 | 100 |
| *V.parahaemolyticus* | 0 | 0 | 0 | 0 | 0 | | 0 | | 6 | 100 |
| Total | 6 | 6 | 6 | 6 | 6 | | 6 | | 6 | 100 |

**Table S5.** Training matrix of fluorescence response pattern obtained from an array of UCNPs@COPs against 7 bacteria (OD_600_=0.1) in physiological saline. LDA was carried out and resulting in 3 factors of the canonical scores and group generation. Jackknifed classification matrix showed the 100% correct classification.

| Analytes  Bacteria | Fluorescence Response Pattern | | |  | Results LDA | | | Group |
| --- | --- | --- | --- | --- | --- | --- | --- | --- |
|  | C1 | C2 | C3 |  | F1 | F2 | F3 |  |
| *E. coli* | 0.269 | 0.468 | 0.369 |  | -25.350 | 2.535 | 4.797 | 1 |
| *E. coli* | 0.265 | 0.463 | 0.362 |  | -25.240 | 2.080 | 4.259 | 1 |
| *E. coli* | 0.249 | 0.463 | 0.353 |  | -28.193 | 1.748 | 3.304 | 1 |
| *E. coli* | 0.249 | 0.451 | 0.345 |  | -26.296 | 1.252 | 2.702 | 1 |
| *E. coli* | 0.247 | 0.450 | 0.342 |  | -26.632 | 1.142 | 2.490 | 1 |
| *E. coli* | 0.245 | 0.447 | 0.330 |  | -26.566 | -0.696 | 1.969 | 1 |
| *S. aureus* | 0.443 | 0.230 | 0.281 |  | 46.347 | -7.002 | 2.278 | 2 |
| *S. aureus* | 0.442 | 0.206 | 0.271 |  | 50.161 | -6.712 | 1.143 | 2 |
| *S. aureus* | 0.438 | 0.205 | 0.271 |  | 49.597 | -6.349 | 0.960 | 2 |
| *S. aureus* | 0.426 | 0.200 | 0.263 |  | 48.463 | -6.171 | 0.040 | 2 |
| *S. aureus* | 0.425 | 0.196 | 0.262 |  | 48.951 | -5.887 | -0.167 | 2 |
| *S. aureus* | 0.416 | 0.195 | 0.261 |  | 47.655 | -5.256 | -0.596 | 2 |
| *Salmonella* | 0.280 | 0.446 | 0.248 |  | -22.599 | -18.156 | 1.085 | 3 |
| *Salmonella* | 0.278 | 0.444 | 0.238 |  | -22.828 | -19.766 | 0.685 | 3 |
| *Salmonella* | 0.262 | 0.441 | 0.231 |  | -25.052 | -19.341 | -0.341 | 3 |
| *Salmonella* | 0.259 | 0.441 | 0.230 |  | -25.612 | -19.247 | -0.504 | 3 |
| *Salmonella* | 0.259 | 0.430 | 0.229 |  | -23.745 | -18.444 | -0.841 | 3 |
| *Salmonella* | 0.257 | 0.424 | 0.223 |  | -23.046 | -18.839 | -1.294 | 3 |
| *L. monocytogenes* | 0.385 | 0.256 | 0.304 |  | 32.003 | -0.332 | 1.216 | 4 |
| *L. monocytogenes* | 0.385 | 0.239 | 0.299 |  | 35.106 | 0.355 | 0.528 | 4 |
| *L. monocytogenes* | 0.370 | 0.233 | 0.299 |  | 33.527 | 1.895 | -0.315 | 4 |
| *L. monocytogenes* | 0.368 | 0.232 | 0.291 |  | 33.116 | 0.748 | -0.670 | 4 |
| *L. monocytogenes* | 0.362 | 0.221 | 0.289 |  | 34.305 | 1.964 | -1.316 | 4 |
| *L. monocytogenes* | 0.356 | 0.215 | 0.284 |  | 34.080 | 2.034 | -1.954 | 4 |
| *C. sakazakii* | 0.237 | 0.378 | 0.303 |  | -15.962 | 1.195 | -1.382 | 5 |
| *C. sakazakii* | 0.233 | 0.374 | 0.300 |  | -15.872 | 1.395 | -1.776 | 5 |
| *C. sakazakii* | 0.231 | 0.373 | 0.292 |  | -16.239 | 0.258 | -2.110 | 5 |
| *C. sakazakii* | 0.230 | 0.367 | 0.281 |  | -15.613 | -1.133 | -2.654 | 5 |
| *C. sakazakii* | 0.227 | 0.360 | 0.279 |  | -14.930 | -0.732 | -3.064 | 5 |
| *C. sakazakii* | 0.217 | 0.355 | 0.270 |  | -15.988 | -0.997 | -3.913 | 5 |
| *V. parahaemolyticus* | 0.223 | 0.378 | 0.349 |  | -16.991 | 10.560 | -0.677 | 6 |
| *V. parahaemolyticus* | 0.218 | 0.374 | 0.344 |  | -17.330 | 10.504 | -1.118 | 6 |
| *V. parahaemolyticus* | 0.218 | 0.370 | 0.343 |  | -16.665 | 10.583 | -1.279 | 6 |
| *V. parahaemolyticus* | 0.218 | 0.370 | 0.328 |  | -17.139 | 7.933 | -1.730 | 6 |
| *V. parahaemolyticus* | 0.216 | 0.363 | 0.325 |  | -16.150 | 8.172 | -2.101 | 6 |
| *V. parahaemolyticus* | 0.213 | 0.356 | 0.323 |  | -15.401 | 8.619 | -2.523 | 6 |
| *S. flexneri* | 0.293 | 0.347 | 0.374 |  | 1.283 | 11.836 | 2.135 | 7 |
| *S. flexneri* | 0.287 | 0.346 | 0.373 |  | 0.363 | 12.279 | 1.804 | 7 |
| *S. flexneri* | 0.274 | 0.340 | 0.373 |  | -0.788 | 13.960 | 1.015 | 7 |
| *S. flexneri* | 0.273 | 0.331 | 0.367 |  | 0.671 | 13.713 | 0.527 | 7 |
| *S. flexneri* | 0.270 | 0.328 | 0.364 |  | 0.651 | 13.790 | 0.242 | 7 |
| *S. flexneri* | 0.257 | 0.318 | 0.357 |  | -0.056 | 14.510 | -0.856 | 7 |

**Table S6.** Fluorescence response patterns using the sensor array against the bacteria mixtures of *E. coli* and *S. aureus* with different ratios (100:0, 80:20, 60:40, 50:50, 40:60, 20:80, and 0:100, respectively) in physiological saline. LDA was carried out and resulting in 3 factors of the canonical scores and group generation. In each case, a bacteria concentration of OD_600_ = 0.1 was used.

| *E. coli* ：*S. aureus* | Fluorescence Response Pattern | | | Results LDA | | | Group |
| --- | --- | --- | --- | --- | --- | --- | --- |
|  | C1 | C2 | C3 | F1 | F2 | F3 |  |
| 0:100 | 0.288 | 0.318 | 0.255 | -10.955 | 0.223 | 1.862 | 1 |
| 0:100 | 0.288 | 0.313 | 0.245 | -11.267 | 0.822 | -0.255 | 1 |
| 0:100 | 0.286 | 0.305 | 0.243 | -10.913 | -0.379 | -0.499 | 1 |
| 0:100 | 0.285 | 0.299 | 0.241 | -10.762 | -1.226 | -0.504 | 1 |
| 0:100 | 0.270 | 0.296 | 0.235 | -14.235 | -0.718 | 1.840 | 1 |
| 0:100 | 0.266 | 0.288 | 0.234 | -14.329 | -1.919 | 2.310 | 1 |
| 80:20 | 0.308 | 0.311 | 0.260 | -5.610 | -1.984 | -1.856 | 2 |
| 80:20 | 0.306 | 0.308 | 0.260 | -5.679 | -2.614 | -1.427 | 2 |
| 80:20 | 0.304 | 0.308 | 0.258 | -6.144 | -2.275 | -1.582 | 2 |
| 80:20 | 0.300 | 0.301 | 0.256 | -6.528 | -3.031 | -1.218 | 2 |
| 80:20 | 0.294 | 0.296 | 0.253 | -7.606 | -3.645 | -0.144 | 2 |
| 80:20 | 0.293 | 0.295 | 0.251 | -7.776 | -3.479 | -0.438 | 2 |
| 60:40 | 0.327 | 0.377 | 0.290 | -5.736 | 5.537 | 0.592 | 3 |
| 60:40 | 0.327 | 0.376 | 0.289 | -5.979 | 5.646 | 0.516 | 3 |
| 60:40 | 0.325 | 0.363 | 0.288 | -5.027 | 3.358 | 0.510 | 3 |
| 60:40 | 0.323 | 0.350 | 0.288 | -4.123 | 0.953 | 0.820 | 3 |
| 60:40 | 0.321 | 0.343 | 0.271 | -5.005 | 2.021 | -2.291 | 3 |
| 60:40 | 0.317 | 0.342 | 0.270 | -5.982 | 2.182 | -1.563 | 3 |
| 50:50 | 0.348 | 0.376 | 0.316 | 0.732 | 1.352 | 1.257 | 4 |
| 50:50 | 0.346 | 0.373 | 0.315 | 0.380 | 1.111 | 1.511 | 4 |
| 50:50 | 0.346 | 0.363 | 0.314 | 1.348 | -0.659 | 1.303 | 4 |
| 50:50 | 0.344 | 0.361 | 0.311 | 0.964 | -0.610 | 0.964 | 4 |
| 50:50 | 0.343 | 0.360 | 0.307 | 0.640 | -0.301 | 0.414 | 4 |
| 50:50 | 0.341 | 0.356 | 0.305 | 0.356 | -0.638 | 0.364 | 4 |
| 40:60 | 0.366 | 0.383 | 0.330 | 4.775 | 0.513 | 0.091 | 5 |
| 40:60 | 0.364 | 0.383 | 0.329 | 4.451 | 0.593 | 0.323 | 5 |
| 40:60 | 0.363 | 0.379 | 0.326 | 4.395 | 0.239 | -0.064 | 5 |
| 40:60 | 0.362 | 0.378 | 0.325 | 4.263 | 0.335 | -0.245 | 5 |
| 40:60 | 0.360 | 0.378 | 0.319 | 3.266 | 1.264 | -1.015 | 5 |
| 40:60 | 0.359 | 0.375 | 0.316 | 3.319 | 1.040 | -1.413 | 5 |
| 20:80 | 0.388 | 0.409 | 0.357 | 9.042 | 1.165 | 0.792 | 6 |
| 20:80 | 0.387 | 0.404 | 0.353 | 9.078 | 0.677 | 0.190 | 6 |
| 20:80 | 0.382 | 0.398 | 0.348 | 8.267 | 0.422 | 0.146 | 6 |
| 20:80 | 0.381 | 0.397 | 0.337 | 7.261 | 1.840 | -1.811 | 6 |
| 20:80 | 0.379 | 0.396 | 0.337 | 6.944 | 1.753 | -1.495 | 6 |
| 20:80 | 0.375 | 0.384 | 0.331 | 6.861 | 0.337 | -1.814 | 6 |
| 100:0 | 0.400 | 0.406 | 0.375 | 13.085 | -2.085 | 1.893 | 7 |
| 100:0 | 0.393 | 0.406 | 0.367 | 11.144 | -0.869 | 1.681 | 7 |
| 100:0 | 0.392 | 0.398 | 0.359 | 11.036 | -1.163 | 0.224 | 7 |
| 100:0 | 0.392 | 0.395 | 0.356 | 11.243 | -1.487 | -0.383 | 7 |
| 100:0 | 0.389 | 0.390 | 0.354 | 10.853 | -1.906 | -0.165 | 7 |
| 100:0 | 0.383 | 0.385 | 0.351 | 9.954 | -2.395 | 0.579 | 7 |

**Table S7**. Fluorescence response pattern obtained from an array of UCNPs@COPs probes against *E. coli* at different concentrations (OD_600_ from 0.5 to 0.05) in physiological saline. LDA was carried out and resulting in 3 factors of the canonical scores and group generation. Jackknifed classification matrix showed the 100% correct classification.

| Concentration (OD_600_) | Fluorescence Response Pattern | | | Results LDA | | | Group |
| --- | --- | --- | --- | --- | --- | --- | --- |
|  | C1 | C2 | C3 | F1 | F2 | F3 |  |
| 0.05 | 0.115 | 0.238 | 0.162 | -32.342 | 3.570 | -1.671 | 1 |
| 0.05 | 0.115 | 0.231 | 0.151 | -32.223 | 2.438 | -3.084 | 1 |
| 0.05 | 0.115 | 0.224 | 0.141 | -32.070 | 1.396 | -4.366 | 1 |
| 0.05 | 0.106 | 0.220 | 0.140 | -33.166 | 2.246 | -3.329 | 1 |
| 0.05 | 0.102 | 0.216 | 0.139 | -33.598 | 2.402 | -2.724 | 1 |
| 0.05 | 0.093 | 0.205 | 0.129 | -34.665 | 2.344 | -2.665 | 1 |
| 0.1 | 0.287 | 0.458 | 0.369 | -13.527 | 7.722 | -0.374 | 2 |
| 0.1 | 0.283 | 0.451 | 0.362 | -13.931 | 7.348 | -0.633 | 2 |
| 0.1 | 0.267 | 0.445 | 0.353 | -15.873 | 8.834 | -0.172 | 2 |
| 0.1 | 0.267 | 0.445 | 0.345 | -15.760 | 8.420 | -1.701 | 2 |
| 0.1 | 0.265 | 0.434 | 0.342 | -16.039 | 7.697 | -0.906 | 2 |
| 0.1 | 0.263 | 0.433 | 0.330 | -16.040 | 7.192 | -2.978 | 2 |
| 0.2 | 0.420 | 0.585 | 0.521 | 1.177 | 6.806 | 2.640 | 3 |
| 0.2 | 0.418 | 0.563 | 0.515 | 1.092 | 4.856 | 3.575 | 3 |
| 0.2 | 0.413 | 0.540 | 0.498 | 0.827 | 2.554 | 2.788 | 3 |
| 0.2 | 0.413 | 0.529 | 0.498 | 0.748 | 1.678 | 3.816 | 3 |
| 0.2 | 0.411 | 0.528 | 0.496 | 0.547 | 1.795 | 3.646 | 3 |
| 0.2 | 0.409 | 0.523 | 0.495 | 0.282 | 1.603 | 4.272 | 3 |
| 0.3 | 0.561 | 0.681 | 0.655 | 17.432 | 0.763 | 4.012 | 4 |
| 0.3 | 0.556 | 0.677 | 0.653 | 16.766 | 1.190 | 4.635 | 4 |
| 0.3 | 0.556 | 0.676 | 0.641 | 16.883 | 0.539 | 2.438 | 4 |
| 0.3 | 0.548 | 0.669 | 0.638 | 16.017 | 0.815 | 3.340 | 4 |
| 0.3 | 0.544 | 0.668 | 0.635 | 15.546 | 1.155 | 3.214 | 4 |
| 0.3 | 0.541 | 0.642 | 0.634 | 15.230 | -0.701 | 5.595 | 4 |
| 0.4 | 0.714 | 0.814 | 0.766 | 35.225 | -4.560 | -2.963 | 5 |
| 0.4 | 0.697 | 0.785 | 0.763 | 33.196 | -4.801 | 0.631 | 5 |
| 0.4 | 0.689 | 0.779 | 0.758 | 32.321 | -4.490 | 0.915 | 5 |
| 0.4 | 0.687 | 0.775 | 0.740 | 32.226 | -5.307 | -1.633 | 5 |
| 0.4 | 0.686 | 0.775 | 0.735 | 32.186 | -5.444 | -2.528 | 5 |
| 0.4 | 0.683 | 0.769 | 0.733 | 31.841 | -5.683 | -2.110 | 5 |
| 0.5 | 0.800 | 0.936 | 0.880 | 44.515 | -1.020 | -1.429 | 6 |
| 0.5 | 0.793 | 0.931 | 0.870 | 43.682 | -0.810 | -2.018 | 6 |
| 0.5 | 0.789 | 0.921 | 0.855 | 43.492 | -2.000 | -3.685 | 6 |
| 0.5 | 0.776 | 0.921 | 0.855 | 41.800 | -0.046 | -2.278 | 6 |
| 0.5 | 0.770 | 0.921 | 0.845 | 41.110 | 0.433 | -3.323 | 6 |
| 0.5 | 0.768 | 0.914 | 0.844 | 40.932 | 0.007 | -2.825 | 6 |

**Table S8**. Fluorescence response pattern obtained from an array of UCNPs@COPs probes against *S. aureus* at different concentrations (OD_600_ from 0.5 to 0.05) in physiological saline. LDA was carried out and resulting in 3 factors of the canonical scores and group generation. Jackknifed classification matrix showed the 100% correct classification.

| Concentration (OD_600_) | Fluorescence Response Pattern | | |  | Results LDA | | | Group |
| --- | --- | --- | --- | --- | --- | --- | --- | --- |
|  | C1 | C2 | C3 |  | F1 | F2 | F3 |  |
| 0.05 | 0.268 | 0.144 | 0.195 |  | -30.406 | -1.899 | 0.422 | 1 |
| 0.05 | 0.266 | 0.143 | 0.195 |  | -30.558 | -1.418 | 0.270 | 1 |
| 0.05 | 0.251 | 0.132 | 0.195 |  | -30.817 | 0.800 | -2.054 | 1 |
| 0.05 | 0.244 | 0.129 | 0.185 |  | -32.125 | 0.698 | -1.194 | 1 |
| 0.05 | 0.243 | 0.128 | 0.182 |  | -32.571 | 0.234 | -0.695 | 1 |
| 0.05 | 0.240 | 0.124 | 0.171 |  | -33.804 | -0.890 | 0.378 | 1 |
| 0.1 | 0.443 | 0.230 | 0.281 |  | -15.391 | -20.870 | 6.972 | 2 |
| 0.1 | 0.442 | 0.206 | 0.271 |  | -15.900 | -24.831 | 6.134 | 2 |
| 0.1 | 0.438 | 0.205 | 0.271 |  | -16.053 | -23.976 | 5.807 | 2 |
| 0.1 | 0.426 | 0.200 | 0.263 |  | -17.261 | -22.933 | 5.639 | 2 |
| 0.1 | 0.425 | 0.196 | 0.262 |  | -17.288 | -23.230 | 5.275 | 2 |
| 0.1 | 0.416 | 0.195 | 0.261 |  | -17.743 | -21.454 | 4.728 | 2 |
| 0.2 | 0.567 | 0.356 | 0.492 |  | 10.646 | -4.487 | -7.887 | 3 |
| 0.2 | 0.564 | 0.345 | 0.485 |  | 10.141 | -6.074 | -8.192 | 3 |
| 0.2 | 0.559 | 0.340 | 0.483 |  | 9.848 | -5.610 | -8.801 | 3 |
| 0.2 | 0.555 | 0.337 | 0.481 |  | 9.561 | -5.321 | -9.069 | 3 |
| 0.2 | 0.548 | 0.336 | 0.476 |  | 8.591 | -4.480 | -8.633 | 3 |
| 0.2 | 0.537 | 0.336 | 0.459 |  | 6.101 | -4.652 | -6.317 | 3 |
| 0.3 | 0.687 | 0.475 | 0.583 |  | 22.594 | -5.798 | -2.387 | 4 |
| 0.3 | 0.682 | 0.471 | 0.579 |  | 22.083 | -5.367 | -2.580 | 4 |
| 0.3 | 0.673 | 0.470 | 0.578 |  | 21.626 | -3.548 | -3.212 | 4 |
| 0.3 | 0.673 | 0.456 | 0.571 |  | 21.268 | -6.146 | -3.443 | 4 |
| 0.3 | 0.673 | 0.451 | 0.571 |  | 21.462 | -6.783 | -4.048 | 4 |
| 0.3 | 0.672 | 0.444 | 0.570 |  | 21.559 | -7.530 | -4.712 | 4 |
| 0.4 | 0.779 | 0.619 | 0.671 |  | 32.081 | 2.402 | 4.281 | 5 |
| 0.4 | 0.771 | 0.615 | 0.669 |  | 31.678 | 3.388 | 3.662 | 5 |
| 0.4 | 0.765 | 0.603 | 0.668 |  | 31.668 | 3.540 | 2.080 | 5 |
| 0.4 | 0.765 | 0.597 | 0.665 |  | 31.563 | 2.374 | 1.866 | 5 |
| 0.4 | 0.753 | 0.585 | 0.662 |  | 31.065 | 3.011 | 0.374 | 5 |
| 0.4 | 0.752 | 0.578 | 0.662 |  | 31.218 | 2.664 | -0.462 | 5 |
| 0.5 | 0.879 | 0.770 | 0.804 |  | 47.014 | 15.814 | 4.404 | 6 |
| 0.5 | 0.877 | 0.767 | 0.804 |  | 46.985 | 15.817 | 4.040 | 6 |
| 0.5 | 0.877 | 0.762 | 0.803 |  | 47.063 | 15.055 | 3.614 | 6 |
| 0.5 | 0.877 | 0.761 | 0.802 |  | 46.951 | 14.964 | 3.696 | 6 |
| 0.5 | 0.868 | 0.747 | 0.791 |  | 45.773 | 13.903 | 3.406 | 6 |
| 0.5 | 0.863 | 0.747 | 0.781 |  | 44.342 | 13.573 | 4.819 | 6 |

**Table S9.** Training matrix of fluorescence response pattern obtained from an array of UCNPs@COPs against 7 bacteria (about 10^6^ cfu mL^-1^) in milk. LDA was carried out and resulting in 3 factors of the canonical scores and group generation. Jackknifed classification matrix showed the 100% correct classification.

| Analytes | Fluorescence Response Pattern | | | Results LDA | | | Group |
| --- | --- | --- | --- | --- | --- | --- | --- |
| Bacteria | C1 | C2 | C3 | F1 | F2 | F3 |  |
| *E. coli* | 0.290 | 0.423 | 0.384 | -17.430 | -0.726 | 8.327 | 1 |
| *E. coli* | 0.287 | 0.420 | 0.364 | -17.482 | 1.463 | 6.826 | 1 |
| *E. coli* | 0.285 | 0.418 | 0.350 | -17.766 | 2.931 | 5.827 | 1 |
| *E. coli* | 0.276 | 0.406 | 0.348 | -17.239 | 1.458 | 5.528 | 1 |
| *E. coli* | 0.276 | 0.395 | 0.346 | -15.296 | 0.820 | 5.285 | 1 |
| *E. coli* | 0.263 | 0.389 | 0.336 | -16.835 | 0.392 | 4.474 | 1 |
| *S. aureus* | 0.389 | 0.171 | 0.286 | 47.468 | 2.055 | 0.034 | 2 |
| *S. aureus* | 0.387 | 0.171 | 0.274 | 47.049 | 3.393 | -0.864 | 2 |
| *S. aureus* | 0.384 | 0.166 | 0.271 | 47.269 | 3.189 | -1.153 | 2 |
| *S. aureus* | 0.383 | 0.158 | 0.270 | 48.594 | 2.592 | -1.296 | 2 |
| *S. aureus* | 0.371 | 0.156 | 0.264 | 46.517 | 2.123 | -1.820 | 2 |
| *S. aureus* | 0.370 | 0.151 | 0.259 | 47.383 | 2.252 | -2.179 | 2 |
| *Salmonella* | 0.191 | 0.364 | 0.246 | -26.933 | 3.702 | -2.737 | 3 |
| *Salmonella* | 0.179 | 0.361 | 0.225 | -28.921 | 5.252 | -4.424 | 3 |
| *Salmonella* | 0.175 | 0.357 | 0.212 | -28.832 | 6.337 | -5.386 | 3 |
| *Salmonella* | 0.171 | 0.355 | 0.211 | -29.423 | 5.859 | -5.464 | 3 |
| *Salmonella* | 0.168 | 0.339 | 0.211 | -26.953 | 4.276 | -5.596 | 3 |
| *Salmonella* | 0.165 | 0.335 | 0.208 | -26.885 | 4.192 | -5.921 | 3 |
| *L. monocytogenes* | 0.372 | 0.283 | 0.303 | 23.936 | 7.089 | 1.955 | 4 |
| *L. monocytogenes* | 0.372 | 0.279 | 0.289 | 24.508 | 8.667 | 0.895 | 4 |
| *L. monocytogenes* | 0.369 | 0.263 | 0.282 | 26.829 | 7.985 | 0.289 | 4 |
| *L. monocytogenes* | 0.345 | 0.259 | 0.280 | 22.840 | 5.623 | -0.047 | 4 |
| *L. monocytogenes* | 0.342 | 0.252 | 0.277 | 23.475 | 5.175 | -0.310 | 4 |
| *L. monocytogenes* | 0.341 | 0.244 | 0.268 | 24.724 | 5.716 | -1.044 | 4 |
| *C. sakazakii* | 0.191 | 0.391 | 0.268 | -31.771 | 3.050 | -0.987 | 5 |
| *C. sakazakii* | 0.191 | 0.375 | 0.262 | -28.828 | 2.451 | -1.483 | 5 |
| *C. sakazakii* | 0.186 | 0.374 | 0.258 | -29.606 | 2.368 | -1.822 | 5 |
| *C. sakazakii* | 0.179 | 0.373 | 0.257 | -30.943 | 1.734 | -1.959 | 5 |
| *C. sakazakii* | 0.175 | 0.372 | 0.255 | -31.488 | 1.642 | -2.108 | 5 |
| *C. sakazakii* | 0.166 | 0.368 | 0.248 | -32.653 | 1.309 | -2.706 | 5 |
| *V. parahaemolyticus* | 0.192 | 0.181 | 0.259 | 6.712 | -12.710 | -3.118 | 6 |
| *V. parahaemolyticus* | 0.186 | 0.179 | 0.255 | 5.908 | -12.977 | -3.437 | 6 |
| *V. parahaemolyticus* | 0.184 | 0.176 | 0.242 | 5.898 | -11.611 | -4.419 | 6 |
| *V. parahaemolyticus* | 0.183 | 0.172 | 0.239 | 6.443 | -11.660 | -4.642 | 6 |
| *V. parahaemolyticus* | 0.176 | 0.161 | 0.234 | 7.081 | -12.364 | -5.175 | 6 |
| *V. parahaemolyticus* | 0.169 | 0.160 | 0.226 | 5.920 | -12.148 | -5.804 | 6 |
| *S. flexneri* | 0.296 | 0.347 | 0.380 | -2.431 | -5.732 | 7.569 | 7 |
| *S. flexneri* | 0.295 | 0.344 | 0.366 | -2.264 | -4.250 | 6.529 | 7 |
| *S. flexneri* | 0.294 | 0.343 | 0.358 | -2.231 | -3.272 | 5.894 | 7 |
| *S. flexneri* | 0.282 | 0.331 | 0.357 | -2.425 | -5.382 | 5.692 | 7 |
| *S. flexneri* | 0.277 | 0.326 | 0.355 | -2.447 | -5.945 | 5.473 | 7 |
| *S. flexneri* | 0.276 | 0.320 | 0.354 | -1.470 | -6.319 | 5.307 | 7 |

**Table S10.** Training matrix of fluorescence response pattern obtained from an array of UCNPs@COPs against 7 bacteria (about 10^6^ cfu mL^-1^) in beef. LDA was carried out and resulting in 3 factors of the canonical scores and group generation. Jackknifed classification matrix showed the 100% correct classification.

| Analytes | Fluorescence Response Pattern | | | Results LDA | | | Group |
| --- | --- | --- | --- | --- | --- | --- | --- |
| Bacteria | C1 | C2 | C3 | F1 | F2 | F3 |  |
| *E. coli* | 0.272 | 0.403 | 0.322 | 15.181 | 2.437 | 1.074 | 1 |
| *E. coli* | 0.272 | 0.395 | 0.316 | 14.094 | 2.228 | 0.729 | 1 |
| *E. coli* | 0.271 | 0.391 | 0.314 | 13.793 | 2.475 | 0.542 | 1 |
| *E. coli* | 0.263 | 0.388 | 0.312 | 14.593 | 3.177 | 0.057 | 1 |
| *E. coli* | 0.253 | 0.387 | 0.306 | 16.309 | 3.311 | -0.519 | 1 |
| *E. coli* | 0.250 | 0.382 | 0.294 | 16.069 | 2.132 | -1.022 | 1 |
| *S. aureus* | 0.372 | 0.284 | 0.260 | -18.502 | -5.448 | 0.767 | 2 |
| *S. aureus* | 0.371 | 0.277 | 0.255 | -19.226 | -5.500 | 0.463 | 2 |
| *S. aureus* | 0.370 | 0.258 | 0.254 | -21.637 | -3.721 | -0.169 | 2 |
| *S. aureus* | 0.366 | 0.253 | 0.232 | -21.728 | -6.471 | -0.891 | 2 |
| *S. aureus* | 0.362 | 0.252 | 0.220 | -21.268 | -7.958 | -1.347 | 2 |
| *S. aureus* | 0.357 | 0.252 | 0.219 | -20.443 | -7.515 | -1.599 | 2 |
| *Salmonella* | 0.283 | 0.374 | 0.270 | 9.209 | -4.144 | -0.287 | 3 |
| *Salmonella* | 0.279 | 0.364 | 0.257 | 8.491 | -4.853 | -1.005 | 3 |
| *Salmonella* | 0.274 | 0.364 | 0.257 | 9.322 | -4.491 | -1.228 | 3 |
| *Salmonella* | 0.261 | 0.357 | 0.256 | 10.660 | -2.842 | -2.002 | 3 |
| *Salmonella* | 0.260 | 0.351 | 0.242 | 10.026 | -4.228 | -2.518 | 3 |
| *Salmonella* | 0.251 | 0.337 | 0.239 | 9.530 | -2.619 | -3.371 | 3 |
| *L. monocytogenes* | 0.365 | 0.275 | 0.322 | -18.238 | 6.137 | 1.432 | 4 |
| *L. monocytogenes* | 0.362 | 0.270 | 0.315 | -18.620 | 5.805 | 1.027 | 4 |
| *L. monocytogenes* | 0.357 | 0.263 | 0.314 | -18.565 | 6.756 | 0.560 | 4 |
| *L. monocytogenes* | 0.350 | 0.255 | 0.307 | -18.512 | 6.997 | -0.116 | 4 |
| *L. monocytogenes* | 0.349 | 0.245 | 0.303 | -19.813 | 7.418 | -0.536 | 4 |
| *L. monocytogenes* | 0.349 | 0.244 | 0.286 | -19.851 | 4.765 | -0.896 | 4 |
| *C. sakazakii* | 0.308 | 0.388 | 0.271 | 6.881 | -7.628 | 1.242 | 5 |
| *C. sakazakii* | 0.306 | 0.384 | 0.269 | 6.554 | -7.505 | 0.992 | 5 |
| *C. sakazakii* | 0.303 | 0.378 | 0.262 | 6.294 | -7.719 | 0.554 | 5 |
| *C. sakazakii* | 0.298 | 0.353 | 0.253 | 3.728 | -6.235 | -0.578 | 5 |
| *C. sakazakii* | 0.294 | 0.353 | 0.252 | 4.342 | -6.165 | -0.767 | 5 |
| *C. sakazakii* | 0.285 | 0.352 | 0.249 | 5.919 | -5.574 | -1.244 | 5 |
| *V. parahaemolyticus* | 0.338 | 0.403 | 0.330 | 3.858 | -2.305 | 4.123 | 6 |
| *V. parahaemolyticus* | 0.326 | 0.398 | 0.322 | 5.298 | -2.171 | 3.315 | 6 |
| *V. parahaemolyticus* | 0.326 | 0.388 | 0.303 | 3.838 | -4.303 | 2.639 | 6 |
| *V. parahaemolyticus* | 0.307 | 0.381 | 0.294 | 6.185 | -3.190 | 1.421 | 6 |
| *V. parahaemolyticus* | 0.304 | 0.376 | 0.292 | 5.951 | -2.692 | 1.118 | 6 |
| *V. parahaemolyticus* | 0.303 | 0.369 | 0.291 | 5.189 | -2.161 | 0.829 | 6 |
| *S. flexneri* | 0.290 | 0.337 | 0.353 | 3.186 | 12.175 | 0.571 | 7 |
| *S. flexneri* | 0.284 | 0.335 | 0.339 | 3.922 | 10.773 | -0.040 | 7 |
| *S. flexneri* | 0.280 | 0.333 | 0.339 | 4.215 | 11.247 | -0.273 | 7 |
| *S. flexneri* | 0.279 | 0.330 | 0.326 | 4.000 | 9.409 | -0.632 | 7 |
| *S. flexneri* | 0.275 | 0.330 | 0.324 | 4.748 | 9.643 | -0.883 | 7 |
| *S. flexneri* | 0.267 | 0.322 | 0.320 | 5.019 | 10.552 | -1.531 | 7 |

**Table S11.** Detection and identification of unknown bacteria samples (about 10^6^ cfu mL^-1^ in tap water). All unknown samples could be assigned to the corresponding LDA group defined by the training matrix. According to the verification, 21 unknown samples were classified, representing an accuracy of 100%.

| Samples  # | Fluorescence Response Pattern | | | Analytes | |
| --- | --- | --- | --- | --- | --- |
|  | C1 | C2 | C3 | Identification | Verification |
| 1 | 0.268 | 0.452 | 0.331 | *E. coli* | *E. coli* |
| 2 | 0.427 | 0.216 | 0.262 | *S. aureus* | *S. aureus* |
| 3 | 0.267 | 0.439 | 0.235 | *Salmonella* | *Salmonella* |
| 4 | 0.358 | 0.218 | 0.287 | *L. monocytogenes* | *L. monocytogenes* |
| 5 | 0.218 | 0.358 | 0.302 | *C. sakazakii* | *C. sakazakii* |
| 6 | 0.218 | 0.357 | 0.327 | *V. parahaemolyticus* | *V. parahaemolyticus* |
| 7 | 0.286 | 0.321 | 0.369 | *S. flexneri* | *S. flexneri* |
| 8 | 0.268 | 0.452 | 0.356 | *E. coli* | *E. coli* |
| 9 | 0.427 | 0.218 | 0.263 | *S. aureus* | *S. aureus* |
| 10 | 0.272 | 0.432 | 0.243 | *Salmonella* | *Salmonella* |
| 11 | 0.378 | 0.233 | 0.303 | *L. monocytogenes* | *L. monocytogenes* |
| 12 | 0.229 | 0.357 | 0.295 | *C. sakazakii* | *C. sakazakii* |
| 13 | 0.220 | 0.377 | 0.343 | *V. parahaemolyticus* | *V. parahaemolyticus* |
| 14 | 0.282 | 0.324 | 0.371 | *S. flexneri* | *S. flexneri* |
| 15 | 0.247 | 0.465 | 0.350 | *E. coli* | *E. coli* |
| 16 | 0.421 | 0.224 | 0.265 | *S. aureus* | *S. aureus* |
| 17 | 0.259 | 0.429 | 0.226 | *Salmonella* | *Salmonella* |
| 18 | 0.385 | 0.221 | 0.290 | *L. monocytogenes* | *L. monocytogenes* |
| 19 | 0.234 | 0.362 | 0.294 | *C. sakazakii* | *C. sakazakii* |
| 20 | 0.218 | 0.377 | 0.337 | *V. parahaemolyticus* | *V. parahaemolyticus* |
| 21 | 0.283 | 0.337 | 0.363 | *S. flexneri* | *S. flexneri* |

**Table S12.** Detection and identification of unknown bacteria samples (about 10^6^ cfu mL^-1^ in milk). All unknown samples could be assigned to the corresponding LDA group defined by the training matrix. According to the verification, 3 unknown samples were misclassified, representing an accuracy of 85.7 %.

| Samples  # | Fluorescence Response Pattern | | | Analytes | |
| --- | --- | --- | --- | --- | --- |
|  | C1 | C2 | C3 | Identification | Verification |
| 1 | 0.283 | 0.400 | 0.346 | *E. coli* | *E. coli* |
| 2 | 0.381 | 0.162 | 0.280 | *S. aureus* | *S. aureus* |
| 3 | 0.171 | 0.369 | 0.256 | *C. sakazakii* | *Salmonella* |
| 4 | 0.366 | 0.277 | 0.275 | *L. monocytogenes* | *L. monocytogenes* |
| 5 | 0.191 | 0.385 | 0.256 | *C. sakazakii* | *C. sakazakii* |
| 6 | 0.168 | 0.176 | 0.249 | *V. parahaemolyticus* | *V. parahaemolyticus* |
| 7 | 0.282 | 0.334 | 0.362 | *S. flexneri* | *S. flexneri* |
| 8 | 0.287 | 0.399 | 0.339 | *E. coli* | *E. coli* |
| 9 | 0.376 | 0.161 | 0.270 | *S. aureus* | *S. aureus* |
| 10 | 0.170 | 0.356 | 0.232 | *Salmonella* | *Salmonella* |
| 11 | 0.362 | 0.256 | 0.287 | *L. monocytogenes* | *L. monocytogenes* |
| 12 | 0.184 | 0.354 | 0.239 | *Salmonella* | *C. sakazakii* |
| 13 | 0.182 | 0.168 | 0.248 | *V. parahaemolyticus* | *V. parahaemolyticus* |
| 14 | 0.282 | 0.360 | 0.371 | *S. flexneri* | *S. flexneri* |
| 15 | 0.281 | 0.399 | 0.345 | *E. coli* | *E. coli* |
| 16 | 0.388 | 0.168 | 0.272 | *S. aureus* | *S. aureus* |
| 17 | 0.175 | 0.357 | 0.234 | *Salmonella* | *Salmonella* |
| 18 | 0.355 | 0.262 | 0.278 | *L. monocytogenes* | *L. monocytogenes* |
| 19 | 0.180 | 0.347 | 0.243 | *Salmonella* | *C. sakazakii* |
| 20 | 0.185 | 0.176 | 0.249 | *V. parahaemolyticus* | *V. parahaemolyticus* |
| 21 | 0.280 | 0.336 | 0.377 | *S. flexneri* | *S. flexneri* |

**Table S13.** Detection and identification of unknown bacteria samples (about 10^6^ cfu mL^-1^ in beef). All unknown samples could be assigned to the corresponding LDA group defined by the training matrix. According to the verification, only 2 of 21 unknown samples were misclassified, representing an accuracy of 90.5 %.

| Samples | Fluorescence Response Pattern | | | Analytes | |
| --- | --- | --- | --- | --- | --- |
| # | C1 | C2 | C3 | Identification | Verification |
| 1 | 0.269 | 0.393 | 0.299 | *E. coli* | *E. coli* |
| 2 | 0.370 | 0.255 | 0.245 | *S. aureus* | *S. aureus* |
| 3 | 0.266 | 0.352 | 0.266 | *Salmonella* | *Salmonella* |
| 4 | 0.363 | 0.252 | 0.316 | *L. monocytogenes* | *L. monocytogenes* |
| 5 | 0.306 | 0.380 | 0.317 | *V. parahaemolyticus* | *C. sakazakii* |
| 6 | 0.295 | 0.385 | 0.269 | *C. sakazakii* | *V. parahaemolyticus* |
| 7 | 0.281 | 0.332 | 0.352 | *S. flexneri* | *S. flexneri* |
| 8 | 0.261 | 0.389 | 0.304 | *E. coli* | *E. coli* |
| 9 | 0.367 | 0.260 | 0.231 | *S. aureus* | *S. aureus* |
| 10 | 0.264 | 0.367 | 0.256 | *Salmonella* | *Salmonella* |
| 11 | 0.359 | 0.247 | 0.303 | *L. monocytogenes* | *L. monocytogenes* |
| 12 | 0.305 | 0.376 | 0.261 | *C. sakazakii* | *C. sakazakii* |
| 13 | 0.328 | 0.391 | 0.308 | *V. parahaemolyticus* | *V. parahaemolyticus* |
| 14 | 0.273 | 0.336 | 0.331 | *S. flexneri* | *S. flexneri* |
| 15 | 0.264 | 0.388 | 0.313 | *E. coli* | *E. coli* |
| 16 | 0.358 | 0.270 | 0.225 | *S. aureus* | *S. aureus* |
| 17 | 0.273 | 0.361 | 0.257 | *Salmonella* | *Salmonella* |
| 18 | 0.362 | 0.249 | 0.305 | *L. monocytogenes* | *L. monocytogenes* |
| 19 | 0.305 | 0.380 | 0.263 | *C. sakazakii* | *C. sakazakii* |
| 20 | 0.321 | 0.396 | 0.302 | *V. parahaemolyticus* | *V. parahaemolyticus* |
| 21 | 0.279 | 0.330 | 0.331 | *S. flexneri* | *S. flexneri* |

**Table S14.** Fluorescence response patterns using the sensor array against the bacteria mixtures of *E. coli* and *S. aureus* with different ratios (100:0, 80:20, 60:40, 50:50, 40:60, 20:80, and 0:100, respectively) in tap water. LDA was carried out and resulting in 3 factors of the canonical scores and group generation. In each case, a bacteria concentration of 10^6^ cfu mL^-1^ was used.

| *E. coli* : *S. aureus* | Fluorescence Response Pattern | | | Results LDA | | | Group |
| --- | --- | --- | --- | --- | --- | --- | --- |
|  | C1 | C2 | C3 | F1 | F2 | F3 |  |
| 0:100 | 0.265 | 0.298 | 0.245 | -11.592 | 3.345 | -0.046 | 1 |
| 0:100 | 0.263 | 0.293 | 0.242 | -12.463 | 3.509 | 0.613 | 1 |
| 0:100 | 0.261 | 0.286 | 0.236 | -13.423 | 2.983 | 1.033 | 1 |
| 0:100 | 0.253 | 0.284 | 0.230 | -13.837 | 2.492 | -0.128 | 1 |
| 0:100 | 0.252 | 0.284 | 0.230 | -13.928 | 2.595 | -0.211 | 1 |
| 0:100 | 0.246 | 0.283 | 0.226 | -14.090 | 2.560 | -1.251 | 1 |
| 80:20 | 0.293 | 0.324 | 0.258 | -7.294 | -0.996 | 0.049 | 2 |
| 80:20 | 0.292 | 0.323 | 0.255 | -7.450 | -1.559 | 0.004 | 2 |
| 80:20 | 0.292 | 0.316 | 0.255 | -8.504 | -0.635 | 1.307 | 2 |
| 80:20 | 0.292 | 0.314 | 0.254 | -8.888 | -0.442 | 1.651 | 2 |
| 80:20 | 0.286 | 0.314 | 0.252 | -8.975 | -0.146 | 0.578 | 2 |
| 80:20 | 0.277 | 0.313 | 0.248 | -9.172 | 0.096 | -0.897 | 2 |
| 60:40 | 0.320 | 0.356 | 0.283 | -2.244 | -1.871 | -0.208 | 3 |
| 60:40 | 0.318 | 0.354 | 0.282 | -2.562 | -1.783 | -0.260 | 3 |
| 60:40 | 0.317 | 0.348 | 0.275 | -3.512 | -2.634 | 0.386 | 3 |
| 60:40 | 0.317 | 0.347 | 0.270 | -3.652 | -4.051 | 0.262 | 3 |
| 60:40 | 0.317 | 0.344 | 0.269 | -4.013 | -4.079 | 0.641 | 3 |
| 60:40 | 0.316 | 0.336 | 0.261 | -5.242 | -5.259 | 1.646 | 3 |
| 50:50 | 0.336 | 0.379 | 0.301 | 1.503 | -2.237 | -1.112 | 4 |
| 50:50 | 0.334 | 0.377 | 0.301 | 1.182 | -1.576 | -1.118 | 4 |
| 50:50 | 0.328 | 0.369 | 0.290 | -0.103 | -3.104 | -1.091 | 4 |
| 50:50 | 0.323 | 0.369 | 0.289 | -0.250 | -2.607 | -1.887 | 4 |
| 50:50 | 0.322 | 0.361 | 0.287 | -1.403 | -1.912 | -0.619 | 4 |
| 50:50 | 0.321 | 0.359 | 0.286 | -1.704 | -1.608 | -0.430 | 4 |
| 40:60 | 0.358 | 0.408 | 0.332 | 6.096 | -0.112 | -1.353 | 5 |
| 40:60 | 0.358 | 0.400 | 0.330 | 4.857 | 0.464 | 0.080 | 5 |
| 40:60 | 0.357 | 0.399 | 0.329 | 4.706 | 0.482 | 0.057 | 5 |
| 40:60 | 0.352 | 0.399 | 0.329 | 4.639 | 0.949 | -0.718 | 5 |
| 40:60 | 0.350 | 0.387 | 0.328 | 2.887 | 2.836 | 1.139 | 5 |
| 40:60 | 0.341 | 0.386 | 0.320 | 2.490 | 1.972 | -0.384 | 5 |
| 20:80 | 0.389 | 0.424 | 0.359 | 8.868 | 1.033 | 2.076 | 6 |
| 20:80 | 0.381 | 0.424 | 0.357 | 8.686 | 1.652 | 0.822 | 6 |
| 20:80 | 0.370 | 0.421 | 0.355 | 8.102 | 3.402 | -0.431 | 6 |
| 20:80 | 0.365 | 0.420 | 0.352 | 7.974 | 3.171 | -1.169 | 6 |
| 20:80 | 0.364 | 0.418 | 0.346 | 7.607 | 1.704 | -1.338 | 6 |
| 20:80 | 0.364 | 0.416 | 0.343 | 7.391 | 1.173 | -1.302 | 6 |
| 100:0 | 0.419 | 0.453 | 0.382 | 13.592 | -0.756 | 2.690 | 7 |
| 100:0 | 0.412 | 0.452 | 0.382 | 13.416 | 0.337 | 1.608 | 7 |
| 100:0 | 0.407 | 0.451 | 0.380 | 13.136 | 0.931 | 1.003 | 7 |
| 100:0 | 0.399 | 0.448 | 0.379 | 12.608 | 2.169 | 0.230 | 7 |
| 100:0 | 0.395 | 0.448 | 0.367 | 12.497 | -1.013 | -1.007 | 7 |
| 100:0 | 0.393 | 0.445 | 0.363 | 12.065 | -1.475 | -0.914 | 7 |

**Table S15.** Fluorescence response patterns using the sensor array against the bacteria mixtures of *E. coli* and *S. aureus* with different ratios (100:0, 80:20, 60:40, 50:50, 40:60, 20:80, and 0:100, respectively) in milk. LDA was carried out and resulting in 3 factors of the canonical scores and group generation. In each case, a bacteria concentration of (about 10^6^ cfu mL^-1^) was used.

| *E. coli* : *S. aureus* | Fluorescence Response Pattern | | | Results LDA | | | Group |
| --- | --- | --- | --- | --- | --- | --- | --- |
|  | C1 | C2 | C3 | F1 | F2 | F3 |  |
| 0:100 | 0.238 | 0.270 | 0.245 | -14.910 | 3.953 | 1.121 | 1 |
| 0:100 | 0.237 | 0.264 | 0.241 | -14.842 | 2.704 | 1.156 | 1 |
| 0:100 | 0.236 | 0.264 | 0.240 | -14.988 | 2.749 | 1.224 | 1 |
| 0:100 | 0.234 | 0.259 | 0.237 | -15.142 | 1.976 | 1.368 | 1 |
| 0:100 | 0.228 | 0.258 | 0.226 | -15.825 | 2.687 | 0.046 | 1 |
| 0:100 | 0.228 | 0.241 | 0.215 | -15.291 | -1.011 | 0.027 | 1 |
| 80:20 | 0.292 | 0.292 | 0.284 | -7.332 | 0.341 | 0.610 | 2 |
| 80:20 | 0.289 | 0.289 | 0.275 | -7.503 | 0.256 | -0.571 | 2 |
| 80:20 | 0.287 | 0.288 | 0.261 | -7.345 | 0.276 | -2.845 | 2 |
| 80:20 | 0.280 | 0.288 | 0.258 | -8.511 | 1.434 | -2.779 | 2 |
| 80:20 | 0.273 | 0.288 | 0.257 | -9.600 | 2.338 | -2.142 | 2 |
| 80:20 | 0.271 | 0.285 | 0.253 | -9.836 | 2.175 | -2.413 | 2 |
| 60:40 | 0.338 | 0.301 | 0.323 | -0.712 | -4.862 | 2.142 | 3 |
| 60:40 | 0.338 | 0.300 | 0.312 | -0.387 | -4.956 | 0.089 | 3 |
| 60:40 | 0.333 | 0.295 | 0.301 | -0.924 | -5.159 | -0.896 | 3 |
| 60:40 | 0.331 | 0.295 | 0.297 | -1.058 | -5.049 | -1.314 | 3 |
| 60:40 | 0.323 | 0.287 | 0.296 | -2.289 | -5.478 | 0.162 | 3 |
| 60:40 | 0.318 | 0.277 | 0.282 | -2.514 | -6.890 | -0.799 | 3 |
| 50:50 | 0.364 | 0.340 | 0.357 | 1.953 | -0.156 | 1.452 | 4 |
| 50:50 | 0.357 | 0.337 | 0.354 | 0.925 | 0.269 | 1.884 | 4 |
| 50:50 | 0.357 | 0.332 | 0.348 | 1.179 | -0.905 | 1.454 | 4 |
| 50:50 | 0.355 | 0.330 | 0.343 | 0.971 | -0.865 | 0.813 | 4 |
| 50:50 | 0.343 | 0.318 | 0.337 | -0.671 | -1.694 | 2.300 | 4 |
| 50:50 | 0.341 | 0.316 | 0.332 | -0.817 | -1.834 | 1.842 | 4 |
| 40:60 | 0.395 | 0.355 | 0.368 | 6.717 | -1.719 | -1.492 | 5 |
| 40:60 | 0.392 | 0.353 | 0.362 | 6.377 | -1.648 | -2.053 | 5 |
| 40:60 | 0.384 | 0.352 | 0.362 | 5.079 | -0.668 | -1.151 | 5 |
| 40:60 | 0.382 | 0.346 | 0.362 | 4.798 | -1.601 | -0.250 | 5 |
| 40:60 | 0.380 | 0.338 | 0.360 | 4.613 | -3.067 | 0.588 | 5 |
| 40:60 | 0.372 | 0.336 | 0.355 | 3.396 | -2.224 | 0.710 | 5 |
| 20:80 | 0.413 | 0.389 | 0.410 | 7.972 | 3.042 | 0.662 | 6 |
| 20:80 | 0.413 | 0.387 | 0.408 | 8.065 | 2.520 | 0.599 | 6 |
| 20:80 | 0.409 | 0.386 | 0.396 | 7.625 | 3.037 | -1.005 | 6 |
| 20:80 | 0.401 | 0.377 | 0.392 | 6.662 | 2.296 | -0.191 | 6 |
| 20:80 | 0.400 | 0.376 | 0.389 | 6.507 | 2.302 | -0.360 | 6 |
| 20:80 | 0.397 | 0.370 | 0.381 | 6.387 | 1.377 | -0.964 | 6 |
| 100:0 | 0.458 | 0.409 | 0.441 | 14.217 | 0.621 | -0.239 | 7 |
| 100:0 | 0.446 | 0.408 | 0.441 | 12.267 | 2.201 | 1.051 | 7 |
| 100:0 | 0.440 | 0.407 | 0.440 | 11.208 | 2.996 | 1.651 | 7 |
| 100:0 | 0.438 | 0.407 | 0.431 | 11.235 | 3.118 | 0.154 | 7 |
| 100:0 | 0.438 | 0.406 | 0.427 | 11.291 | 3.092 | -0.592 | 7 |
| 100:0 | 0.434 | 0.399 | 0.418 | 11.052 | 2.029 | -1.050 | 7 |

**Table S16.** Fluorescence response patterns using the sensor array against the bacteria mixtures of *E. coli* and *S. aureus* with different ratios (100:0, 80:20, 60:40, 50:50, 40:60, 20:80, and 0:100, respectively) in beef. LDA was carried out and resulting in 3 factors of the canonical scores and group generation. In each case, a bacteria concentration (about 10^6^ cfu mL^-1^) was used.

| *E. coli* : *S. aureus* | Fluorescence Response Pattern | | | Results LDA | | | Group |
| --- | --- | --- | --- | --- | --- | --- | --- |
|  | C1 | C2 | C3 | F1 | F2 | F3 |  |
| 0:100 | 0.266 | 0.291 | 0.250 | -9.436 | -2.805 | -2.101 | 1 |
| 0:100 | 0.265 | 0.277 | 0.235 | -10.563 | -2.960 | 0.939 | 1 |
| 0:100 | 0.263 | 0.276 | 0.234 | -10.721 | -2.879 | 0.731 | 1 |
| 0:100 | 0.258 | 0.272 | 0.229 | -11.261 | -2.948 | 0.471 | 1 |
| 0:100 | 0.249 | 0.261 | 0.228 | -11.213 | -4.951 | -0.344 | 1 |
| 0:100 | 0.245 | 0.259 | 0.222 | -11.873 | -4.526 | -0.461 | 1 |
| 80:20 | 0.309 | 0.330 | 0.278 | -6.559 | 0.341 | 0.650 | 2 |
| 80:20 | 0.306 | 0.330 | 0.277 | -6.698 | 0.437 | 0.196 | 2 |
| 80:20 | 0.302 | 0.330 | 0.276 | -7.061 | 0.735 | -0.779 | 2 |
| 80:20 | 0.289 | 0.326 | 0.268 | -8.253 | 1.390 | -2.382 | 2 |
| 80:20 | 0.286 | 0.323 | 0.265 | -8.539 | 1.274 | -2.538 | 2 |
| 80:20 | 0.282 | 0.317 | 0.260 | -9.036 | 1.052 | -2.332 | 2 |
| 60:40 | 0.338 | 0.360 | 0.315 | -2.411 | -0.480 | 0.041 | 3 |
| 60:40 | 0.338 | 0.358 | 0.311 | -2.894 | -0.024 | 0.711 | 3 |
| 60:40 | 0.337 | 0.351 | 0.309 | -2.814 | -1.090 | 1.552 | 3 |
| 60:40 | 0.325 | 0.343 | 0.297 | -4.268 | -0.696 | 0.882 | 3 |
| 60:40 | 0.324 | 0.335 | 0.295 | -4.082 | -2.119 | 1.654 | 3 |
| 60:40 | 0.319 | 0.334 | 0.292 | -4.587 | -1.627 | 1.067 | 3 |
| 50:50 | 0.367 | 0.387 | 0.335 | -0.408 | 1.560 | 1.725 | 4 |
| 50:50 | 0.364 | 0.385 | 0.329 | -1.136 | 2.323 | 2.181 | 4 |
| 50:50 | 0.354 | 0.382 | 0.328 | -1.405 | 1.772 | 0.088 | 4 |
| 50:50 | 0.353 | 0.379 | 0.326 | -1.588 | 1.562 | 0.491 | 4 |
| 50:50 | 0.353 | 0.368 | 0.322 | -1.453 | -0.136 | 2.098 | 4 |
| 50:50 | 0.349 | 0.367 | 0.322 | -1.607 | -0.294 | 1.247 | 4 |
| 40:60 | 0.401 | 0.436 | 0.386 | 4.593 | 2.137 | -1.260 | 5 |
| 40:60 | 0.400 | 0.435 | 0.384 | 4.422 | 2.205 | -1.219 | 5 |
| 40:60 | 0.392 | 0.425 | 0.376 | 3.551 | 1.909 | -1.134 | 5 |
| 40:60 | 0.392 | 0.415 | 0.373 | 3.758 | 0.164 | 0.137 | 5 |
| 40:60 | 0.383 | 0.415 | 0.366 | 2.584 | 1.456 | -1.140 | 5 |
| 40:60 | 0.382 | 0.414 | 0.359 | 1.640 | 2.893 | -0.300 | 5 |
| 20:80 | 0.443 | 0.478 | 0.420 | 7.986 | 4.917 | 0.146 | 6 |
| 20:80 | 0.439 | 0.476 | 0.403 | 5.808 | 7.927 | 1.419 | 6 |
| 20:80 | 0.431 | 0.472 | 0.402 | 5.659 | 7.140 | 0.236 | 6 |
| 20:80 | 0.425 | 0.465 | 0.401 | 5.735 | 5.654 | -0.590 | 6 |
| 20:80 | 0.414 | 0.454 | 0.390 | 4.478 | 5.580 | -0.693 | 6 |
| 20:80 | 0.413 | 0.447 | 0.389 | 4.689 | 4.224 | -0.070 | 6 |
| 100:0 | 0.470 | 0.488 | 0.472 | 14.917 | -3.363 | -0.502 | 7 |
| 100:0 | 0.467 | 0.473 | 0.470 | 15.353 | -6.327 | 0.480 | 7 |
| 100:0 | 0.456 | 0.472 | 0.468 | 14.854 | -6.312 | -1.839 | 7 |
| 100:0 | 0.455 | 0.470 | 0.462 | 14.248 | -5.704 | -0.991 | 7 |
| 100:0 | 0.452 | 0.465 | 0.454 | 13.316 | -4.962 | -0.430 | 7 |
| 100:0 | 0.452 | 0.457 | 0.442 | 12.274 | -4.449 | 1.964 | 7 |

**Table S17. Comparison of different detection methods**

| Methods | Limit of detection  (cfu ml^-1^) | Multiplexing ability | Sample source | Acquisition time (h) | Reference |
| --- | --- | --- | --- | --- | --- |
| Multiplex PCR | 10 | 6 | Clinical specimens | 5 | [[4](#_ENREF_4)] |
| Multiplex Real-time PCR | 10^3^ | 3 | Milk | 8 | [[5](#_ENREF_5)] |
| Multiplex PCR | 10^3^ | 3 | Milk | 3 | [[6](#_ENREF_6)] |
| Polymer/Peptide complex sensor array | 10^6^ | 14 | Urine | 1 | [[7](#_ENREF_7)] |
| CDs sensor array | 10^9^ | 6 | Tap water | 1 | [[8](#_ENREF_8)] |
| AIE sensor array | 10^8^ | 8 | - | 0.5 | [[9](#_ENREF_9)] |
| UCNPs@COPs  sensor array | 10^6^ | 7 | Tap water  Milk  Beef | 0.25  0.5  1 | This work |

**Reference:**

1. Yin M, Wu C, Li H, Jia Z, Deng Q, Wang S, Zhang Y. Simultaneous Sensing of Seven Pathogenic Bacteria by Guanidine-Functionalized Upconversion Fluorescent Nanoparticles. *ACS Omega* 2019; 4:8953-8959.

2. Zheng; Z, Guo; J, Mao; H, Xu; Q, Qin; J, Yan. F. Metal-Containing Poly(ionic liquid) Membranes for Antibacterial Applications. *ACS Biomaterials Science & Engineering* 2017.

3. Wang K, Li Y, Li H, Yin M, Liu H, Deng Q, Wang S. Upconversion fluorescent nanoparticles based-sensor array for discrimination of the same variety red grape wines. *RSC Advances* 2019; 9:7349-7355.

4. Duan QQ, Lu SQ, Hu YX, Shen SN, Xi BS, Wang XN, Sun WP. A Multiplex PCR Assay Mediated by Universal Primer for the Diagnosis of Human Meningitis Caused by Six Common Bacteria. *Russian Journal of Genetics* 2018; 54:423-430.

5. Jeong YS, Jung HK, Hong J-H. Multiplex real-time polymerase chain reaction for rapid detection of Staphylococcus aureus, Vibrio parahaemolyticus, and Salmonella typhimurium in milk and kimbap. *Journal of the Korean Society for Applied Biological Chemistry* 2013; 56:715-721.

6. Wei C, Zhong J, Hu T, Zhao X. Simultaneous detection of Escherichia coli O157:H7, Staphylococcus aureus and Salmonella by multiplex PCR in milk. *3 Biotech* 2018; 8.

7. Han J, Cheng H, Wang B, Braun MS, Fan X, Bender M, Huang W, Domhan C, Mier W, Lindner T, et al. A Polymer/Peptide Complex-Based Sensor Array That Discriminates Bacteria in Urine. *Angew Chem Int Ed Engl* 2017; 56:15246-15251.

8. Zheng L, Qi P, Zhang D. Identification of bacteria by a fluorescence sensor array based on three kinds of receptors functionalized carbon dots. *Sensors and Actuators B: Chemical* 2019; 286:206-213.

9. Liu GJ, Tian SN, Li CY, Xing GW, Zhou L. Aggregation-Induced-Emission Materials with Different Electric Charges as an Artificial Tongue: Design, Construction, and Assembly with Various Pathogenic Bacteria for Effective Bacterial Imaging and Discrimination. *ACS Appl Mater Interfaces* 2017; 9:28331-28338.
